# Neural Adaptation at Stimulus Onset and Speed of Neural Processing as Critical Contributors to Speech Comprehension Independent of Hearing Threshold or Age

**DOI:** 10.3390/jcm13092725

**Published:** 2024-05-06

**Authors:** Jakob Schirmer, Stephan Wolpert, Konrad Dapper, Moritz Rühle, Jakob Wertz, Marjoleen Wouters, Therese Eldh, Katharina Bader, Wibke Singer, Etienne Gaudrain, Deniz Başkent, Sarah Verhulst, Christoph Braun, Lukas Rüttiger, Matthias H. J. Munk, Ernst Dalhoff, Marlies Knipper

**Affiliations:** 1 Department of Otolaryngology, Head and Neck Surgery, University of Tübingen, Elfriede-Aulhorn-Str. 5, 72076 Tübingen, Germany; jakob.schirmer@student.uni-tuebingen.de (J.S.); stephan.wolpert@med.uni-tuebingen.de (S.W.); kodap95@gmail.com (K.D.); moritz.ruehle@student.uni-tuebingen.de (M.R.); wertz.jakob@gmx.de (J.W.); therese.eldh@uni-tuebingen.de (T.E.); katharina.bader@med.uni-tuebingen.de (K.B.); wibke.singer@uni-tuebingen.de (W.S.); lukas.ruettiger@uni-tuebingen.de (L.R.); 2 Department of Biology, Technical University Darmstadt, 64287 Darmstadt, Germany; 3 Department of Information Technology, Ghent University, Technologiepark 126, 9052 Zwijnaarde, Belgium; marjoleen.wouters@ugent.be (M.W.); sarah.verhulst@ugent.be (S.V.); 4 Lyon Neuroscience Research Center, Centre National de la Recherche Scientifique UMR5292, Inserm U1028, Université Lyon 1, Centre Hospitalier Le Vinatier-Bâtiment 462–Neurocampus, 95 Boulevard Pinel, 69675 Bron CEDEX, France; e.p.c.gaudrain@rug.nl; 5 Department of Otorhinolaryngology, University Medical Center Groningen (UMCG), Hanzeplein 1, BB21, 9700 RB Groningen, The Netherlands; d.baskent@rug.nl; 6 Magnetoencephalography-Centre and Hertie Institute for Clinical Brain Research, University of Tübingen, Otfried-Müller-Straße 27, 72076 Tübingen, Germany; christoph.braun@med.uni-tuebingen.de; 7 Center for Mind and Brain Research, University of Trento, Palazzo Fedrigotti-corso Bettini 31, 38068 Rovereto, Italy; 8 Department of Psychiatry & Psychotherapy, University of Tübingen, Calwerstraße 14, 72076 Tübingen, Germany

**Keywords:** cochlear synaptopathy, presbycusis, cochlear amplifier, OLSA, ASSR

## Abstract

**Background**: It is assumed that speech comprehension deficits in background noise are caused by age-related or acquired hearing loss. **Methods**: We examined young, middle-aged, and older individuals with and without hearing threshold loss using pure-tone (PT) audiometry, short-pulsed distortion-product otoacoustic emissions (pDPOAEs), auditory brainstem responses (ABRs), auditory steady-state responses (ASSRs), speech comprehension (OLSA), and syllable discrimination in quiet and noise. **Results**: A noticeable decline of hearing sensitivity in extended high-frequency regions and its influence on low-frequency-induced ABRs was striking. When testing for differences in OLSA thresholds normalized for PT thresholds (PTTs), marked differences in speech comprehension ability exist not only in noise, but also in quiet, and they exist throughout the whole age range investigated. Listeners with poor speech comprehension in quiet exhibited a relatively lower pDPOAE and, thus, cochlear amplifier performance independent of PTT, smaller and delayed ABRs, and lower performance in vowel-phoneme discrimination below phase-locking limits (/o/-/u/). When OLSA was tested in noise, listeners with poor speech comprehension independent of PTT had larger pDPOAEs and, thus, cochlear amplifier performance, larger ASSR amplitudes, and higher uncomfortable loudness levels, all linked with lower performance of vowel-phoneme discrimination above the phase-locking limit (/i/-/y/). **Conslusions**: This study indicates that listening in noise in humans has a sizable disadvantage in envelope coding when basilar-membrane compression is compromised. Clearly, and in contrast to previous assumptions, both good and poor speech comprehension can exist independently of differences in PTTs and age, a phenomenon that urgently requires improved techniques to diagnose sound processing at stimulus onset in the clinical routine.

## 1. Introduction

Age-related hearing loss is the most prevalent disorder of aging and is associated with future cognitive impairment [1]. Recent studies indicate that an association between hearing and cognition also exists in individuals with subclinical hearing loss—that is, those with normal pure-tone audiograms below 25 dB hearing level up to 4 kHz [2,3]. A deterioration in speech recognition over the lifespan is also observed, despite unchanged pure-tone thresholds [3].

This suggests that the cognitive decline after hearing loss or the worsening of speech comprehension is not necessarily linked with differences in the pure-tone audiogram as currently measured in the clinical routine. Afferent auditory fiber loss (cochlear synaptopathy) may be a candidate contributor that can precede loss of outer hair cells (OHCs) and an overt threshold loss, as shown in animals [4,5,6] and predicted for humans [7,8,9,10,11,12,13,14]. As such, the so-called ‘hidden hearing loss’ refers specifically to the damage to low spontaneous rate (SR) high-threshold auditory nerve fibers (ANFs) that are said to play a role in coding supra-threshold sound features of speech in noise [6,11,12,15,16,17,18,19]. Thereby compromising temporal envelope (TENV) coding, the phase-locked neural response of a population of peripheral and brainstem neurons to a stimulus envelope [20]. In accordance with that, subjects with hidden hearing loss and with speech comprehension deficits in noise display normal pure-tone and speech audiometry thresholds in quiet, and well-synchronized ABRs [21,22]. This form of cochlear synaptopathy has to be separated from auditory neuropathy spectrum disorder of syndromic or non-syndromic origin, which develops in early childhood and leads to the structural anomalies of cochlear nerve hypoplasia or aplasia [22,23] or from postoperative ANF damage during acoustic neuroma surgery [24,25]. In both cases, ABR peak amplitudes and speech audiometry in quiet and, thus, speech recognition below the phase-locking limit (PLL), which is encoded as temporal fine-structure (TFS) by the ANF, are severely affected in adulthood [22,23]. Besides speech comprehension deficits with a major focus on speech-intelligibility deficits in noise [26,27,28,29], to date, variations in speech comprehension deficits in quiet that cannot be explained by hereditary or postoperative causes are less understood [28]. They are in all cases explained by an increase in the hearing thresholds, and therefore predicted to be compensated for by an increase in loudness. To gain a deeper insight into the causes of speech comprehension deficits, which may possibly exist or worsen independently of clinically normal hearing thresholds [3,30], we have used a combination of measurement methods that are not necessarily used in clinical routine. Thus, PTTs, as typically implemented in clinical audiometry, effectively integrate signals over ~500 ms, and thus reflect the adapted state of nerve firing that follows an overshoot of the discharge rate at the onset of the stimuli [31]. Here, we measured PTTs in a total of 89 young, middle-aged, and older individuals for four different frequency ranges: pure-tone averages (PTA) of low frequencies (i) “PTA-LF” (0.125–1 kHz); (ii) “PTA4” (0.5–4 kHz), which are mostly measured to evaluate the hearing threshold in clinical otolaryngology [32,33]; (iii) high frequencies “PTA-HF” (6–10 kHz) to go beyond the frequencies of 6 kHz that are typically deemed sufficient to intelligibly convey speech in communication systems [34] and that still cover conventional frequency ranges measured in many clinical studies [32,35]; and (iv) extended high frequencies “PTA-EHF” (11.2–16 kHz), which have been assumed to play a decisive role in improved spatial hearing [36].

We combined these measures with the analysis of speech reception thresholds (SRTs) in quiet to reflect the state of audibility, and, in noise, to test for the capacity for speech discrimination. For the measurement of SRT_50_ in quiet, or at a fixed ipsilateral or contralateral noise level, we used the standard German Matrix test Oldenburger Satztest (OLSA) for either unfiltered “broadband” speech (OLSA-BB); low-pass filtered speech (OLSA-LP), frequency components above 1.5 kHz deleted from the OLSA power spectrum, leaving available TFS cues [37]; and high-pass filtered speech (OLSA-HP), below 1.5 kHz deleted from the OLSA power spectrum, leaving available TENV cues [38,39], as previously described [40]. To evaluate the residual speech comprehension performance independent of PTTs, we next subtracted the three OLSA threshold predictions from the three measured OLSA thresholds and averaged them, using a multivariate regression model based on principal component analysis (PCA). We thereby classified subjects with matched PTTs into groups with good, standard, and poor speech comprehension.

Considering factors that possibly contribute to differences independent of PTT, analysis procedures were chosen that enable the diagnosis of signal transmission at stimulus onset prior to auditory nerve firing-rate adaptations that are reached >500 ms. This early stage of sound transmission around the stimulus onset is not reflected in PTTs. Here, as a metric for the temporal precision of auditory coding, the so-called ASSRs were used, which reflect phase-locked neural activity to periodic stimuli coded by TENV [41]. Carrier tones at 4 and 6 kHz were used to induce ASSRs that were modulated at 116 Hz, the same frequency that was used as the fundamental frequency for the speech stimuli. This was at the same time a modulation frequency that is expected to generate ASSR responses by subcortical rather than cortical components [42,43].

In addition, pDPOAE growth functions were measured in all young, middle-aged, and older individuals, a measure that also identifies pre-neural input signals to the inner hair cells (IHCs) prior to firing-rate adaptation [44]. Indeed, in contrast to PTTs, pDPOAEs reflect the state of the cochlear amplifier with high accuracy by using stimulus pulse widths that are more than an order of magnitude lower than PTTs [39,40,44].

Finally, we analyzed peak amplitudes of ABRs, the short-latency evoked potentials that emanate from the auditory pathways and nuclei of the brain stem, and develop within the first 10 ms of stimulation. This also enables the precise detection of signal transmission at stimulus onset. Here, click-induced, supra-threshold auditory brainstem responses (amplitudes and latencies) of ABR wave I/II (generated by the auditory nerve and dorsal cochlear nucleus) [45,46], ABR wave III (generated by the superior olivary complex (SOC) and lateral lemniscus) [47], and ABR wave V and VI (generated by the inferior colliculus (IC) [48] and the medial geniculate body (MGB) [49] were specifically detected as described [50,51]. Finally, considering that language comprehension is dependent upon the correct discrimination of vowels [52] and consonants [53], which, in turn, requires precise TFS coding (below the human PLL, i.e., below 1.5 k Hz) and TENV coding (above the PLL, above 1.5 kHz) [54,55], we hoped to be able to link differences in speech comprehension in subjects of different ages or PTTs with differential contributions of ANFs to TNF or TENV coding, as previously suggested [56].

Toward this aim, we used decomposed narrowband signals that varied depending on whether nerve discharges were above the PLL as /i/-/y/ and /di/-/bi/ phoneme pairs or below the PLL as /o/-/u/ and /du/-/bu/ phoneme pairs [40,57].

Strikingly, we found that differences in signal transmission at the onset of the stimulus contributed to differences in speech comprehension in quiet and noise, independent of age or threshold. This underlines the high relevance of fast transmission speed of auditory information at the beginning of the stimulus for speech comprehension in human clinical studies, as previously speculated on, based on a numerical model [58]. We may thus have identified a new cause of speech-discrimination disorders that has to date evaded typical diagnostic procedures.

## 2. Materials and Methods

The study was conducted at the Department of Otolaryngology of the University of Tübingen and approved by the ethics committee of Tübingen University (Faculty of Medicine; ethical approval-number 392/2021BO2). Written informed consent was given by all participants. All methods followed the Declaration of Helsinki by the World Medical Association (WMA) for human research ethics.

### 2.1. Participants

We recruited 112 participants aged between 18 and 76 years. A checklist to inquire about any comorbidities was used for study exclusion. Among these were as follows: other hearing-related conditions such as tinnitus, or previous ear surgery, as well as systemic diseases known to affect hearing. Ultimately, 89 participants were included in the analysis, and the remaining ones were excluded due to comorbidities (threshold elevation beyond 40 dB hearing loss in one or more frequencies and tinnitus) or because of lacking compliance. These 89 participants were evenly distributed across three age groups, young (18–29 years, n = 29), middle-aged (30–55 years, n = 32), and older (56–76 years, n = 28) (Supplementary Table S1). Participants’ age, gender, handedness, and confirmation of normal middle ear function by tympanometry are provided (Supplementary Table S1). Of the 89 participants, only 63 could be measured in quiet, ipsilateral, and contralateral noise conditions of the German word matrix test OLSA, leaving 26 participants who were only tested in the quiet condition.

### 2.2. Neuropsychiatric Scores

As an exclusion criteria, we applied two validated questionnaires: “Becks Depression Inventory II” (BDI), shown to screen for depression in a clinical setting [59] and the Geriatric Depression Scale (GDS)**,** with a focus on affective and cognitive domains [60] to exclude depression and a German version of the Mini-Mental State Examination (MMSE) [61,62] to exclude dementia. In this test, the participants answer questions related to orientation in space and time, word short-term memory, subtracting, attentive listening, spelling, reading, writing, executive tests, and visuo-construction. The self-assessment of hearing ability was analyzed using an adapted questionnaire that assessed hearing ability in various conversational situations, also concerning education level [63].

### 2.3. Otoscopy and Impedance Audiometry

The ear examination was carried out by ENT physicians from the Department of Otolaryngology, Head and Neck Surgery at the University of Tübingen. Tympanometry and stapedial-reflex measurements were performed using an AT235 (Interacoustics, Middelfart, Denmark) tympanometry system using a 226 Hz stimulus to ensure intact middle-ear transmission [64] and generally intact neural pathways [65].

### 2.4. Pure-Tone Audiometry

Using an AT 1000 Audiometer (Auritec, medizindiagnostische Geräte Gmbh, Hamburg, Germany), PTTs were measured for air and bone conduction, as well as the uncomfortable loudness level (UCL). Bone conduction at 0.25, 0.5, 1, 1.5, 2, 4, and 6 kHz was measured using a B71 bone transducer (Radioear, Middelfart, Denmark). The default pure-tone audiometric thresholds from 0.125 to 10 kHz, and the UCL (0.25, 0.5, 1, 2, 4, and 6 kHz) were measured using Beyerdynamic AT1350A on-ear headphones (Beyerdynamic, Heilbronn, Germany). In addition, EHF thresholds were measured using Sennheiser HDA300Pro (Sennheiser, Wedemark-Wennebostel, Germany) on-ear headphones at the frequencies 11.2, 12.5, 14, and 16 kHz. The Sennheiser HDA300Pro achieves a nominal level of 123 dB SPL (6 Hz to 23 kHz). All measurements were conducted in a sound-attenuating chamber (Industrial Acoustics Company GmbH, Niederkrüchten, Germany).

PTA of low frequencies (PTA-LF; 0.125, 0.25, 0.5, and 1 kHz), high frequencies (PTA-HF; 6, 8, and 10 kHz), extended high frequencies (PTA-EHF; 11.3, 12.5, 14, and 16 kHz), and PTA4 (0.5, 1, 2, and 4 kHz) were derived from the right-ear thresholds.

### 2.5. Auditory Brainstem Responses (ABRs)

The ABR measurements were performed monaurally using three electrodes (Neuroline 720, Ambu, Bad Nauheim, Germany), with electrode impedance consistently below 2 kΩ (ground: Fpz—above the nasion; reference—inverting input (−): Fz—hairline; non-inverting input (+): mastoid). As an amplifier, the actiCHamp Plus64 (Brain Products GmbH, Gilching, Germany) was set up according to the manufacturer’s specifications and at a sampling rate of 50 kHz. Acoustic click stimuli (83 µs) were presented at two different stimulus levels (70 dB SPL and 80 dB SPL) with 3000 repetitions of alternating polarity. Stimuli were generated using a Scarlet Focusrite 8i8 gen 3 (Focusrite, UK) soundcard and presented through ER2 transducers and disposable ER1-14A earpieces (Etymotic Research, Elk Grove Village, IL, USA). To minimize muscle effects, the participants lay on their backs during the measurements. ER2 in-ear loudspeakers exhibit frequency bandwidth limits to approximately 8 kHz, which allowed conclusions to be drawn about changes caused by the frequency content within this bandwidth.

After band-pass filtering (30–2000 Hz; first order FIR filter, Hamming windowed), ABR waveform components were averaged at each stimulus level. Wave V was determined to be the most prominent peak, typically appearing 5–6 ms after stimulus onset. Waves I, II, III, and VI were then assigned to peaks at 1 to 2 ms, 2 to 3 ms, 3 to 4 ms, and 6 to 7 ms after stimulus onset, respectively. Wave amplitudes were calculated in µV as the difference between leading positive and trailing negative deflections/peaks, as previously described [50,51]. Their latency was measured from the leading positive peak.

### 2.6. Auditory Steady-State Response (ASSR)

ASSR was measured using the same recording setup and without changing the position of the participants. The modulation frequency was set to 116 Hz (rectangular 100% amplitude modulation as described in [66]) and two blocks of 800 epochs each were recorded at carrier frequencies of 4 and 6 kHz at 70 dB SPL rms. The stimulus duration was set to 400 ms, with an epoch duration of 500 ± 10 ms. Responses from all epochs were averaged and the spectral power was calculated by FFT (MATLAB 2021b). ASSR peak amplitudes (µV) were averaged for the first three harmonics [66]. Measurements with inadequate signal-to-noise ratios (SNR below 2) or ASSR peak amplitudes higher than 0.15 µV were excluded from the statistical evaluation.

### 2.7. Distortion-Product Otoacoustic Emissions (DPOAEs)

Input–Output (I/O) functions of pDPOAEs were measured to characterize the pre-neural state of the cochlea. Using a pulsed waveform for the second primary (f_2_), along with onset decomposition [44], a technique to capture the short-latency nonlinear-distortion (ND) component of the DPOAE [67], artefactual interference effects from the longer-latency component can be safely avoided [68]. From these pDPOAE I/O functions, two measures were analyzed in the results section: The extrapolated pDPOAE threshold and the acceptance rate. When stimulus levels of both primaries are chosen according to a so-called scissors paradigm [69], extrapolated DPOAE thresholds (level of the estimated distortion product threshold; L_EDPT_) based on semi-logarithmically scaled I/O functions have been shown to correlate nearly 1:1 with the pure-tone threshold for hearing losses up to about 50 dB [44,70,71,72]. Most previous studies, such as Kummer 1998 [69], Boege 2002 [70], Georga 2003 [71] and Johnson 2007 [72]) used several criteria for I/O function acceptance; these effectively avoid hard-to-interpret DPOAE I/O functions that lead to large extrapolation errors. Thus, the acceptance rate is the number of I/O functions passing these criteria divided by the number of measurements. The acceptance rate informs about DPOAE levels (because any pDPOAE value to be included in the extrapolation procedure must have an SNR of 10 dB or above), as well as the integrity of the measured I/O function. In the present study, pDPOAE I/O functions were measured using an in-ear-probe at 8 frequencies (f_2_ = 0.8, 1.2, 1.5, 2, 3, 4, 6, and 8 kHz) using an adaptive algorithm comprising at least four pDPOAE values. For details of recording and method, see Supplementary Material, section “pDPOAE measurements”.

### 2.8. Speech Reception Thresholds (OLSA)

Speech intelligibility was tested using the “Oldenburger Satz Test” (OLSA), the German version of the International Matrix test [73,74], applying three different configurations of the masker noise presentation in each of the three different speech material filtering conditions. The three noise conditions were no noise (quiet), ipsilateral noise, and contralateral noise.

The three speech material filtering conditions were as follows: unfiltered broadband speech (OLSA-BB), low-pass filtered speech (OLSA-LP, components above 1.5 kHz were deleted from the OLSA power spectrum), and high-pass filtered speech (OLSA-HP, components below 1.5 kHz were deleted from the OLSA power spectrum) (see for details [40]). The nine conditions were presented in random order. Sentences consisted of five words, with a name, verb, number, adjective, and object, and each keyword having ten response possibilities, producing a large number of (10^5^) combinations from an inventory of a total of 50 words. The speech material of the OLSA is spoken by a male speaker [73], the average F0 of which we determined to be 116 Hz. For each condition, participants were presented with 20 sentences. As initial training, an OLSA-BB and OLSA-HP test of 20 sentences each was completed before starting the target sentence presentations. This served to reduce the impact of the training effect, which has been shown to be the largest after the first presentation (approx. 1 dB, [75]). In order to reduce fatigue due to the extent of speech testing, the session was paused after the first three OLSA-BB, OLSA-HP, and OLSA-LP in quiet conditions with an intermittent request, during which psychoacoustic tasks were performed, before it continued for contralateral and ipsilateral noise conditions. The target sentences and the masker noise were presented monaurally (speaker always right, masker noise presented to the same or the other ear) over ER2 transducers (see Section 2.5, ABR). The level of the target sentence varied and was decreased after a correct response (i.e., increasing difficulty) or increased after an incorrect response (i.e., decreasing difficulty). The masker noise was derived from the speech material by randomly shifted overlapping and, thus, exhibits the same long-term spectrum [73], and closely resembles spectra of several speech materials in other languages [76,77]. The level of the masker noise was fixed at 70 dB SPL. Speech reception thresholds for 50% correctly identified words (SRT_50_) were determined for the three noise masker conditions [40]. For each of the nine different conditions, blocks of 20 sentences were presented.

### 2.9. Pure-Tone-Normalized OLSA Threshold

This study was particularly focused on factors beyond pure-tone thresholds and how they may relate to OLSA thresholds. To evaluate the role of these other factors, OLSA thresholds were quantitatively normalized for PTTs of all available frequencies (0.125–16 kHz). This correction was calculated independently for the quiet, ipsilateral noise, and contralateral noise conditions by performing a multivariate regression between all three OLSA (BB, LP, and HP) thresholds and the first five principal components (MatLab Version 2021b) of all audiometric thresholds; the latter to avoid overfitting. The PCA was performed by employing a singular-value decomposition algorithm. Together, the first five PCA components captured 93% of the variations in audiometric thresholds. OLSA predictors for each individual subject were derived by evaluating the linear regression model using these first five components of the pure-tone thresholds of each participant. Thus, 77.8% of the participants with good, standard, and poor speech comprehension in contralateral noise were grouped into the same categories in the speech-in-quiet tasks. The same can only be said for 39.9% of participants from the speech comprehension in ipsilateral noise tasks, meaning that subjects with good or poor speech comprehension, independent of PTT in quiet and age, were not necessarily those that exhibited good or poor speech comprehension independent of PTT in ipsilateral noise conditions and age.

To evaluate the residual speech comprehension performance, we subtracted the three OLSA threshold predictions from the three measured OLSA thresholds and averaged them. This average value will be referred to as the PT-normalized OLSA threshold (PNOT). Based on PNOT, the cohort was divided into three equally sized groups with “good”, “normal”, and “poor” speech performance. We verified that this data-driven approach resulted in groups with matched average pure-tone audiometry thresholds within ± 4.5 dB for the four PTA frequency ranges (PTA-LF, PTA4, PTA-HF, PTA-EHF).

### 2.10. Stimuli for Phoneme Discrimination

The stimuli used for the phoneme discrimination task were computer generated from recordings from a male speaker, and using analysis/re-synthesis as implemented in the WORLD vocoder [78]. To match the OLSA speech material, their average fundamental frequency (F0) was set to 116 Hz during synthesis. A total of eight phonemes, two pairs of steady-state vowels, and two pairs of consonant–vowel syllables were used as stimuli.

The vowel pairs /o/ (like in *oder*, “or” in German) and /u/ (like in *Du*, “you” in German) that differed in their first formant (F1, see Supplementary Table S2) and are located well *below* the supposed PLL in humans (~1.5 kHz) were synthesized with a 30 ms raised cosine ramp at the onset and offset, and had a total duration of approximately 414 ms (corresponding to 48 F0 cycles). Similarly, the /du/-/bu/ syllable pair only differed at frequencies below the PLL, and only within the first 100 ms. The following 371 ms were exactly identical between the two syllables. In addition, the vowel segment of this syllable pair, /u/, was identical to the isolated steady-state /u/ used in the /o-u/ vowel pair, except that it was trimmed such that the overall duration of the syllables was 471 ms.

The vowel pairs /i/ (like in *sie*, “she” in German) and /y/ (like in *üben,* “practice” in German) only differed in their second and third formants (F2 and F3), which were *above* the PLL (see Supplementary Table S2). As a result, it is expected that encoding of this /i/-/y/ contrast could not rely on TFS, but rather on envelope coding. This /i/-/y/ vowel contrast had the same durations and ramps as the other vowel pair described above. The /di/-/bi/ syllable pair was also built to only differ in frequencies above the PLL, and within the spectral power of the first 100 ms. Again, the /i/ from these syllables was identical in spectral shape to the /i/ used in the vowel pair /i/-/y/. All stimuli were then spectrally tilted to ensure similar signal-to-noise ratios above and below the PLL when presented in the speech-shaped noise used in the OLSA task.

From each of the four stimulus pairs, a nine-step continuum was generated by gradually modifying the formant’s frequencies on a log-frequency scale. Following piloting, a large and a small contrast were selected for each pair. Given the large inter-individual variability observed during piloting, this selection aimed to ensure that floor or ceiling effects would be avoided for at least one of these contrast magnitudes.

The stimuli, presented via ER2 earphones, were equalized such that the average level of the stimuli belonging to a given continuum was the same for all pairs, and was adjusted to a 60 dB SPL energy-equivalent continuous sound level (Leq). However, minor level fluctuations within a continuum were preserved to ensure that the level of the formants that remained identical throughout the continuum were not affected. Calibration was performed using a B&K Type 4157 Microphone (Hottinger Brüel & Kjær, Virum, Denmark) in combination with an artificial ear with a volume of 1 cm^3^ and a 20 s integration time.

### 2.11. Behavioral Phoneme Discrimination Task 

The phoneme discrimination between the pairs (/o/-/u/, /i/-/y/, /du/-/bu/, /di/-/bi/) was measured using a three-alternative forced choice (3AFC) paradigm. For each phoneme pair, we measured two difficulty levels, easy and difficult. We quantified the difference using nine levels that were tested in pilot experiments from which two pairs, one easy and one difficult, were selected for the psychoacoustic session. The differences in difficulty level, e.g., /du/-/bu/ in the easy condition was eight out of nine, while in the difficult condition, the difference was only four out of nine. The respective other level differences for the difficult and easy conditions were for /di/-/bi/ eight and four, for /o/-/u/ four and two, and for /i/-/y/ three and one. Together with the three different noise conditions (quiet, ipsilateral, and contralateral noise), we acquired data under a total of six conditions. Each condition was repeated nine times, producing a total of 54 trials. The noise was the same speech-shaped noise used during the OLSA measurement and presented at 0 dB SNR.

To minimize learning effects, conditions were randomly reordered at the beginning of the measurement. As a short initial training run, three trials of phoneme discrimination of the four syllable pairs were performed.

The right ear was used to test each syllable pair, using the same transducers as used for the OLSA test. Before each condition of the test, the participants were given four training trials with visual feedback. However, responses from this training were not included in the data analysis. During the main test, participants did not receive any feedback on the correctness of their responses.

### 2.12. Statistical Analysis

To test for the significance of group differences, statistical tests for non-normally distributed data were applied. ABR wave amplitudes and latencies were compared using one-way analysis of variance (ANOVA) for group differences. The resulting *p*-values smaller than the criterion of α = 0.05 were considered statistically significant. The correlation of two measurement parameters was verified by the Pearson correlation coefficient (r). ASSR amplitudes (µV) were compared by Mann–Whitney U tests between good and poor performers, between poor and standard performers, and between standard and good performers (1-sided hypothesis).

### 2.13. Pure-Tone-Normalized OLSA (PNOT)

For PNOT, the score of percent-correct answers was compared by Mann–Whitney U tests between good and poor performers, between poor and standard performers, and between standard and good performers (1-sided hypothesis: The group of poor/standard performers contains more participants with low-percentage correct scores relative to the standard/good performers. A resulting *p*-value equal to or smaller than α = 0.05 was considered statistically significant and noted as an asterisk in the respective figure panel (Supplementary Figure S1; Supplementary Table S4). A *p*-value smaller than 0.1 was noted with an asterisk in brackets to indicate a trend in the distribution, though not reaching statistical significance. Statistical comparisons were made for the percent-correct scores obtained for the “difficult” discrimination task (small spectral and temporal syllable contrast), and for the scores obtained for the “easy” discrimination task (larger spectral and temporal syllable contrast).

### 2.14. Variance Analysis

Analysis of variance for speech perception thresholds beyond pure-tone thresholds was performed by least-square multivariate linear fitting of the five added principal components (PCs) derived from pure-tone thresholds, and one additional observable that was tested for its contribution to total speech comprehension variance. To ensure the uniqueness of the multivariate linear model, we first removed all linear correlations between the five PCs and the tested observable. This can be understood as removing the influence of PTTs on ABR wave amplitude or latency or other parameters such as L_EDPT_ thresholds, ASSR amplitudes, or phoneme discrimination. An inherent risk of the increase in dimensions of the regression model is the possibility of overfitting. To eliminate this effect, we compared the observed increase in explainable variance in the observed six-dimensional model to the variance of 10,000 pseudo models in which we randomly shuffled the additional observable before fitting the model. This gave us a reliable estimate of what gain in the explained variance was achieved based on chance. The results are presented as stacked bar diagrams showing the percentage of variance that could be attributed to each of the observables (Supplementary Figure S1, Supplementary Table S3). To better illustrate the magnitude of the effect, we additionally computed the standard deviation in the unit of the SRT (dB), which can be attributed to the new observable, by taking the ratio of the variance of the observable to the overall variance, and multiplying by the standard deviation (SD) of the sample in (dB) (Supplementary Figure S1; Supplementary Table S3). However, during this computation, we assumed a normal distribution of the five PCs and the additional tested observable, an assumption that is not required for the statistical evaluation based on permutation analysis.

### 2.15. Data Distributions

If not indicated otherwise, data are presented as group mean and SD for the number of participants or ears (*n*), as specified in the figure legends. For visualization of the different performances of poor, good, and standard speech reception, syllable discrimination performance (% correct) was classified in histograms with logarithmic class sizes.

## 3. Results

From all 89 young, middle-aged, and older participants (Supplementary Table S1), a five-grade custom questionnaire for subjective self-evaluation of hearing performance (excellent, very good, good, moderate, bad) in different conversational situations was conducted, and three psychometric tests (BDI, GDS, MMSE) were performed to exclude any confounding, severe psychiatric factors such as depression or dementia onset (Supplementary Table S1). There were no confounding abnormalities identified in the included group.

### 3.1. Pure-Tone Thresholds Are Elevated with Age

**Figure 1:**
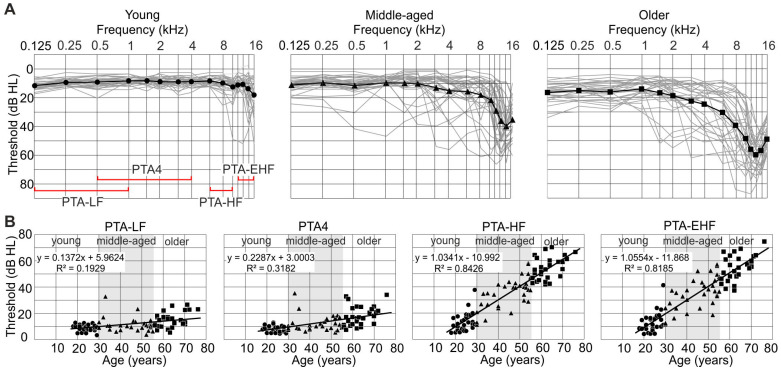
Elevated hearing thresholds correlated with age, in particular at high frequencies: (**A**) Individual (grey lines) and mean (black line) PTTs for the three age groups young (left), middle-aged (center), and older (right) used for PTAs of four different frequency ranges: low frequencies “PTA-LF” (0.125–1 kHz), main-language region “PTA4” (0.5–4 kHz), high frequencies “PTA-HF” (6–10 kHz), and extended high frequencies “PTA-EHF” (11.2–16 kHz), illustrated in red on the abscissa of the left-most audiogram. The group mean thresholds are plotted in black (young: circles; middle-aged: triangles; older: squares). (**B**) Scatterplots for individual hearing thresholds as a function of age, split into the four PTA frequency ranges. The shaded area delineates the age range of the middle-aged group. *p*-Values (Pearson’s correlation): p(PTA-LF) = 0.000016; p(PTA4) < 0.00001; p(PTA-HF) < 0.00001, p(PTA-EHF) < 0.00001.

As described in Section 2.4, PTTs were collected for frequencies between 0.125 and 16 kHz. The distinct frequency ranges PTA-LF [0.125–1 kHz], PTA4 [0.5–4 kHz), and PTA-HF [6–10 kHz], and extended high frequencies PTA-EHF [11.2–16 kHz] are illustrated on the abscissa of the leftmost audiogram (Figure 1A). Comparison of the three age groups revealed group differences that shifted toward significantly elevated thresholds above 8 kHz, which, as reported in previous studies, was particularly prominent for EHF thresholds between 11.2 and 16 kHz (Figure 1A) [79,80,81,82].

In addition, all four PTAs correlated significantly with age (Figure 1B, PTA-LF: *p* = 0.000016, R^2^ = 0.1929; PTA4: *p* < 0.00001, R^2^ = 0.3182; PTA-HF: *p* < 0.00001, R^2^ = 0.8426; PTA-EHF: *p* < 0.00001, R^2^ = 0.8185). The slope of the regression lines (see Figure 1B, R^2^ values) is much steeper if the correlation is computed with respect to the HF and EHF averages, with considerably higher R^2^ values than for the lower-frequency averages (0.84 and 0.81 as compared to 0.19 and 0.32 for PTA-LF and PTA4, Figure 1B). This supports the notion that age-dependent hearing loss is predominantly an increasing loss of high-frequency hearing.

### 3.2. Speech Reception Thresholds Elevate with PTA-Threshold and Age

**Figure 2:**
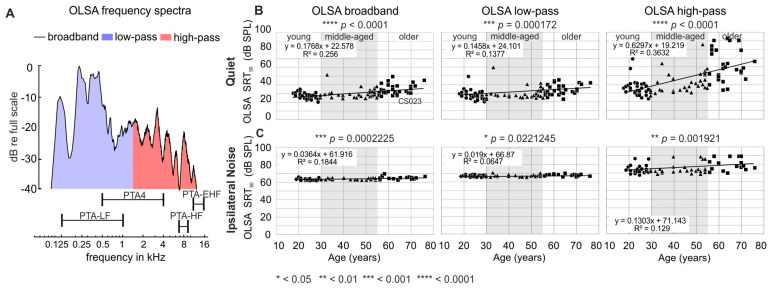
(**A**) Power spectrum of the OLSA speech material (broadband, black curve), of high-pass filtered speech (OLSA-HP, red shaded area), and low-pass filtered speech (OLSA-LP, blue shaded area), shown peak-normalized to 0 dB and 1/f-corrected. The four different PTA frequency ranges are depicted: low frequencies “PTA-LF” (0.125–1 kHz), “PTA4” (0.5–4 kHz), high frequencies “PTA-HF” [6–10 kHz], and extended high frequencies “PTA-EHF” (11.2–16 kHz). (**B**,**C**) The influence of noise on OLSA SRT_50_ was examined using differently filtered speech material. Noise conditions in (**B**) quiet and (**C**) ipsilateral noise. Columns provide results for broadband and filtered OLSA stimuli. OLSA SRTs are provided as a function of age.

SRT_50_ were next analyzed using either an unfiltered speech-in-noise signal (OLSA-BB), a low-pass filtered condition (OLSA-LP), or a high-pass filtered condition (OLSA-HP, see Section 2.8 and Figure 2A). SRT_50_ were determined for OLSA-BB, OLSA-LP, and OLSA-HP (Figure 2B,C) and plotted as a function of age. We observed that SRT_50_ in quiet were significantly positively correlated with age, with the strongest dependence for the high-pass filtered condition, as shown for quiet (Figure 2B, OLSA-BB: R^2^ = 0.256, *p* < 0.00001; OLSA-LP: R^2^ = 0.138, *p* = 0.000172; OLSA-HP: R^2^ = 0.363, *p* < 0.00001).

SRT_50_ under speech-shaped ipsilateral noise were also significantly correlated with age, but the dependence on age was markedly reduced (Figure 2C, OLSA-BB: R^2^ = 0.184, *p* = 0.000223; OLSA-LP: R^2^ = 0.065, *p* = 0.0221; OLSA-HP: R^2^ = 0.129, *p* = 0.001921), and the respective slopes were shallower than the slopes for the speech-in-quiet condition (Figure 2C).

To summarize: SRT_50_, as analyzed using defined OLSA filter conditions, revealed that speech comprehension significantly depended on age under all conditions. The variance and regression curve increased most with OLSA-HP, when frequencies below 1.5 kHz were deleted from the speech power spectra. This is surprising, considering the low age-dependent threshold increase in the range of these low-frequency spectra.

### 3.3. The Supra-Threshold ABR Wave Decreases with Elevated, Age-Dependent PTA-EHF

**Figure 3:**
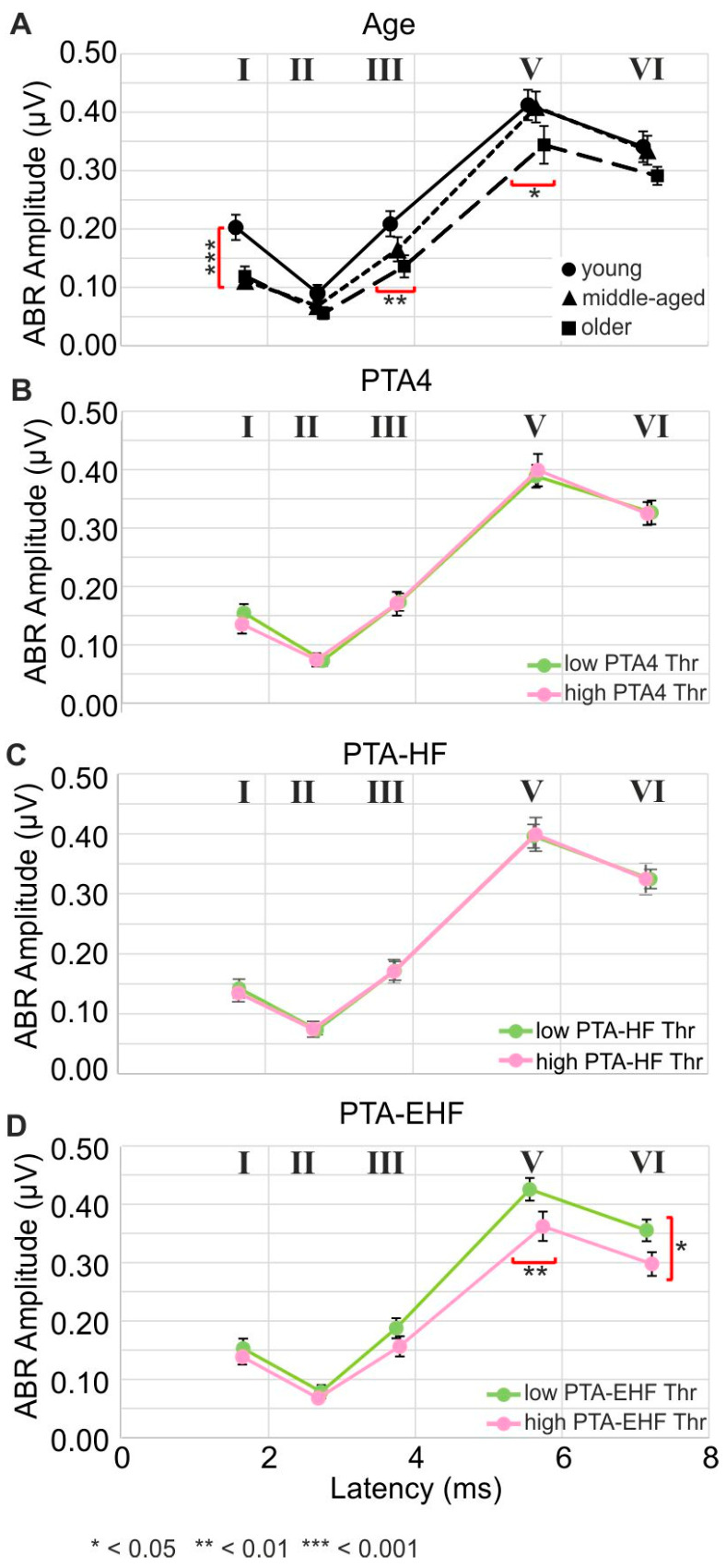
ABR as a function of age, pure-tone averages, and speech comprehension: (**A**) ABR wave amplitude and latencies grouped by age. Circles represent young, triangles middle-aged, and squares older participants. (**B**–**D**) ABR wave amplitudes and latencies grouped for participants with low (green) and high (pink) thresholds of PTA4 (**B**), PTA-HF (**C**), and PTA-EHF (**D**).

Supra-threshold ABR waves (amplitudes and latencies) of ABR wave I–VI were measured by acoustic stimulation at 80 dB SPL rms in young, (Figure 3A, solid line, circle), middle-aged (Figure 3A, dotted line, triangle), and older participants (Figure 3A, dotted line, square) using loudspeakers with bandwidth, limited to ~8 kHz (see Section 2.5). ABR wave I had significantly lower amplitude in the middle-aged and older participants than in the young (Figure 3A, young: 0.203 µV, n = 27; middle-aged: 0.111 µV; n = 27; older: 0.119 µV; n = 19; *p* = 0.00029). However, this lower input was somewhat compensated for by ABR wave VI amplitudes in the middle-aged group (Figure 3A, young: 0.341 µV, n = 27; middle-aged: 0.335 µV, n = 31; older: 0.291, n = 20, *p* = 0.35). Nonetheless, it was still significantly delayed in the older group for wave III and wave V (Figure 3A, wave III, young: 3.67 ms, n = 29; middle-aged: 3.77 ms; n = 31; older: 3.86 ms, n = 24; *p* = 0.0095; wave V, young: 5.55 ms, n = 29; middle-aged: 5.66 ms, n = 32; older: 5.76 ms, n = 25; *p* = 0.018). Interestingly, the lower-amplitude and delayed ABR wave VI in the older group was neither linked to PTT differences in the PTA-4 frequency range (Figure 3B) nor to PTT differences in the PTA-HF frequency range (Figure 3C). When ABR amplitudes were plotted as a function of latencies for PTA-EHFs frequency ranges, altered late ABR wave amplitudes in the older (Figure 3A) were linked to lower and delayed ABR wave V/VI (Figure 3D).

To summarize: Poorer PTA-EHF thresholds as a function of age are associated with a persistently reduced and delayed click-evoked ABR wave V/VI. This strongly indicates that the elevation of PTA-EHF with age may negatively influence both late ABR wave V/VI peak amplitudes and latencies in lower frequency ranges, as also suggested in previous studies [79].

### 3.4. Speech Comprehension Exhibits Components That Are Dependent on and Independent of Pure-Tone Threshold and Age

**Figure 4:**
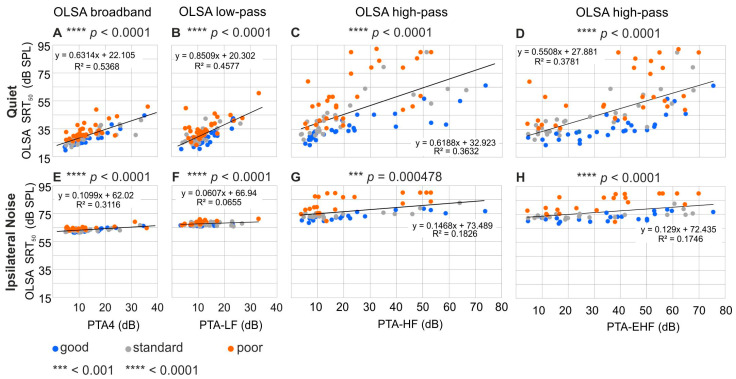
OLSA speech reception threshold SRT_50_ (dB SPL; *y*-axes) for differently filtered OLSA stimuli ((**A**,**E**) broadband, (**B**,**F**) low pass, and (**C**,**D**,**G**,**H**) high-pass) as a function of PTA4 (**A**,**E**), PTA-LF (**B**,**F**), PTA-HF(**C**,**G**), and PTA-EHF (**D**,**H**) (*x*-axes). (**A**–**D**) provide results obtained in quiet (n = 89), (**E**–**H**) under ipsilateral (n = 63) noise condition. Regression lines are plotted in black and include y-intersections and R^2^ values. The different colors assign each subject to one of the three speech comprehension groups: good (blue), standard (grey), and poor (orange).

To investigate the dependence of frequency-specific filtered speech bands on the pure-tone threshold in more detail, we next tested the dependence of all OLSA-SRTs (BB, LP, HP) on their corresponding pure-tone averages, such as PTA-LF, PTA-4, PTA-HF, and PTA-EHF in quiet (Figure 4A–D) and ipsilateral noise conditions (Figure 4E–H, Table 1). In all 12 comparisons, OLSA SRT_50_ significantly depended on the corresponding PTA measure, with stronger scatter for the dependence of OLSA-HP SRT_50_ on PTA-HF, and OLSA-HP on PTA-EHF, under all three noise conditions (Figure 4, Table 1). For OLSA in ipsilateral noise, the correlation shows a clearly reduced slope of the regressions under all frequency conditions, with a fairly homogenous distribution around the regression line for hearing losses up to 40 dB, as shown in previous studies [83].

Under all conditions, the OLSA SRT_50_ correlated better (higher R^2^) with the PTA (Figure 4, Table 1) than with age (Figure 2). This effect was very clear for OLSA-BB in quiet (Figure 4A), with a mean R^2^ = 0.5368 for PTA (Figure 4A) vs. R^2^ = 0.26 for age (Figure 2B). This effect was less pronounced for OLSA-BB in ipsilateral noise with R^2^ = 0.31 for PTA4 (Figure 4E, Table 1) vs. R^2^ = 0.18 for age (Figure 2C), where it almost vanished only for OLSA-LP vs. PTA-LF (Figure 4 and Figure 2).

To examine the variance of OLSA-SRT with the distinct PTA in more detail, we performed a post hoc classification based on PNOT, as described in Section 2.9. This approach was used to separate the cohort into three groups, with a maximum spread in their speech comprehension (Figure 4, see blue = “good” and orange = “poor” dots), as well as the “standard” group with comprehension performance between good and poor groups (Figure 4, grey dots). As expected, participants with poor, standard, and good speech comprehension based on PNOT showed a well-matched mean PTT (Table 1). Interestingly, they were almost the same mean age (Table 1), although the standard PNOT group was on average slightly younger, and exhibited slightly, but not significantly, better PTA-EHF thresholds: PNOT-quiet: p(age) = 0.100; p(PTA-EHF) = 0.150; PNOT-ipsi: p(age) = 0.713; p(PTA-EHF) = 0.547 (Table 1).

Speech-in-quiet comprehension, used here essentially as a control, depended strongly on the PTT, with a slope of more than 0.6 dB/dB. When removing the effect of the PTT upon the PNOT (see Section 2, Supplementary Figure S1, Supplementary Table S3), the multivariate regression of the PTT on OLSA thresholds returned to R^2^ = 0.49.

The remaining variance of the speech intelligibility that was not explained by the PTT comprised in OLSA-BB was 38.7%, corresponding to an SD of 3.7 dB in SRT_50_. In contrast, broadband speech-in-noise comprehension depended on the PTT, with a slope of approximately 0.1 dB/dB, leaving 51.4% of the variance unexplained when removing the effect of the PTT, corresponding to an SD of 1.0 dB in SRT_50_.

In summary: Analyzing the PNOT, we identified good and poor speech comprehension independent of PTT and age. To explain the remaining variance of OLSA-SRT independent of PTT, we (i) compared subjective speech understanding dependent on age with that obtained independent of PTT, and compared good and poor PNOT groups using diagnostic tools that enabled us to identify the efficiency of signal transmission at stimulus onset not reflected in PTTs (see Introduction) as (ii) ASSRs, (iii) precise measurements of the cochlear amplifier using pDPOAEs, (iv) click-evoked central supra-threshold ABRs, and (v) phoneme discrimination ability. The respective shares of the contributions to variance in speech comprehension of each of these measurements are depicted in Supplementary Figure S1 and calculated as the percentage of significant contributions to the variance of OLSA SRT_50_ in Supplementary Table S3.

### 3.5. Differences between Good and Poor Pure-Tone-Normalized OLSA Thresholds (PNOTs) Is a Better Indicator of Self-Assessed Hearing Ability than Age

**Figure 5:**
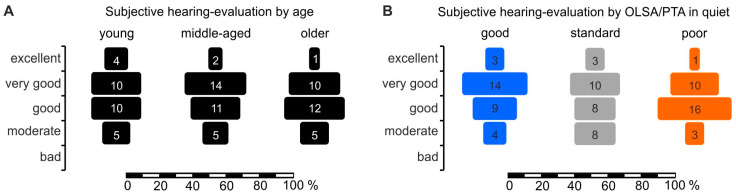
Subjective hearing evaluation by age and speech comprehension: (**A**) shows age groups and (**B**) groups according to objective speech comprehension performance based on OLSA thresholds corrected by PNOTs. *y*-axis: subjective evaluation, *x*-axis: percentage of all responses given by all participants in age groups (**A**) and in PNOT groups (**B**). Participants were asked to rate their hearing as excellent, very good, good, moderate, or bad (*y*-axis labels).

When comparing the self-evaluation of speech comprehension of young, middle-aged, and older participants (Figure 5A), with that obtained when participants are grouped by good, standard, and poor speech comprehension according to PNOTs (Figure 5B), we found PNOT classification of participants to be a better predictor of the subjective assessment of their hearing than their age. In particular, a higher percentage of the middle-aged and older participants rated themselves as hearing very well—comparable to the young population—when the sub-division was assessed according to age (Figure 5A, young, n = 29, middle-aged, n = 32, older n = 28, *p* = 0.50, one-sided Fisher Exact Probability Test for “very good” and “good” assessments). However, when patients were instead classified as having good, standard, or poor speech comprehension by the PNOTs in quiet, we found that the expected self-rated decrease in hearing ability with age was much more congruent with real hearing performance, though not reaching statistical significance (Figure 5B, good, n = 30, standard. n = 29, poor, n = 30, *p* = 0.10, one-sided Fisher Exact Probability Test for “very good” and “good” assessments). There were no statistically significant differences when grouped for PNOTs in ipsilateral noise (good, standard, poor, n = 21, *p* = 0.50).

In summary: The differences between good and poor speech comprehension that remained when OLSA thresholds were normalized for PTTs was a better indicator of self-assessed hearing ability than age, highlighting the relevance of the factors that may contribute to good and poor speech comprehension independent of PTT.

### 3.6. The Difference between Good and Poor PNOTs Shows Low Dependence on Temporal Envelope (TENV) Coding (ASSR)

**Figure 6:**
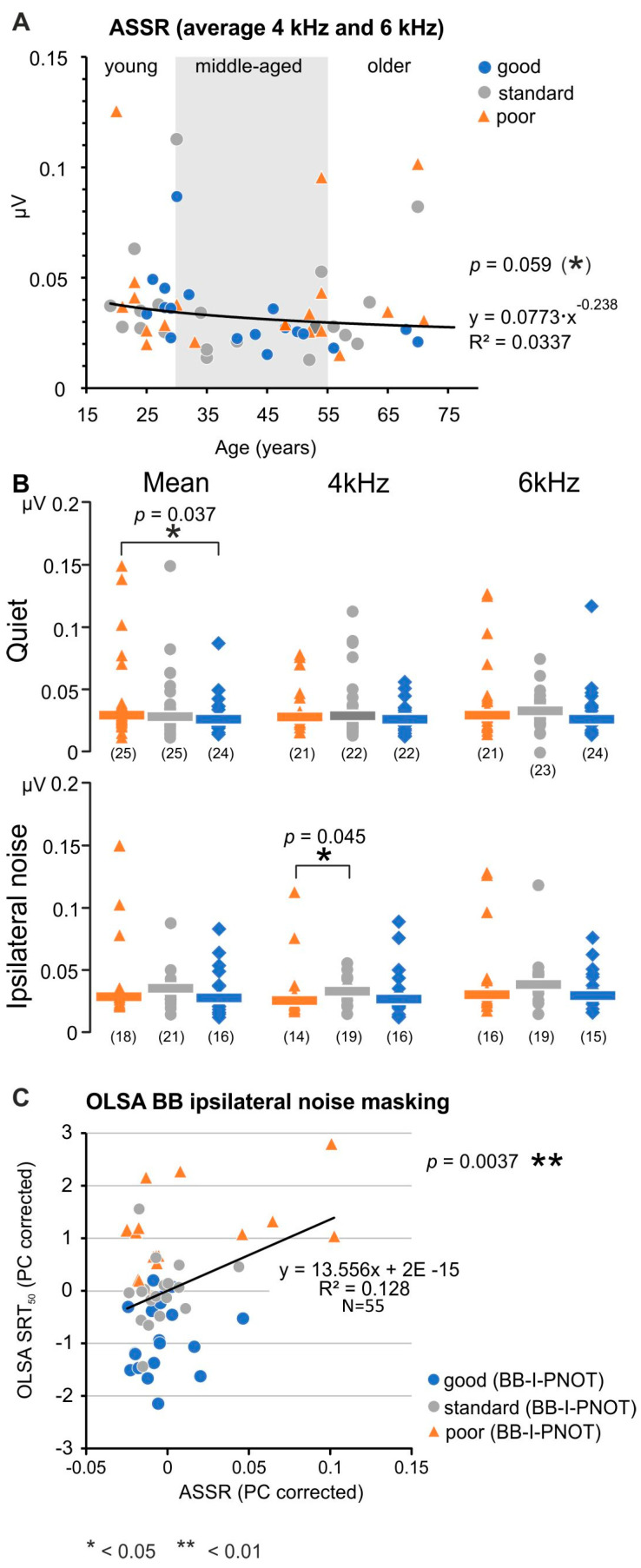
(**A**) ASSR response amplitudes in µV averaged for 4 and 6 kHz carriers as a function of participant age in years. The blue, grey, and orange-colored symbols refer to the good, standard, and poor speech comprehension groups, respectively. (**B**) Median (horizontal bar) and individual participants (symbols) ASSR amplitude averaged for 4 and 6 kHz carriers (Mean, left), 4 kHz carrier (middle), and 6 kHz carrier (right) for the quiet listening condition (upper row), or in ipsilateral noise (lower row). Numbers in brackets indicate the number of participants included in the analyses. (**C**) Regression line (black) of the dependence of OLSA SRT_50_ in ipsilateral noise on ASSR amplitudes (averaged for 4 and 6 kHz carriers) normalized for PTT. The y-intersection, R^2^ value, and *p*-value of regression are given close to the trend line.

The averaged ASSR amplitudes were analyzed next as a function of age at both 4 and 6 kHz carrier frequencies (Figure 6A), and inspected for significance in groups with poor, standard, and good speech comprehension in quiet and in ipsilateral noise (Figure 6B) conditions. A tendency of lower amplitude with increasing age was noted for ASSR amplitudes at both 4 and 6 kHz carrier frequencies (shown for the average of 4 kHz and 6 kHz ASSR responses in Figure 6A), with nearly equal contribution of poor or good PNOTs along the different age.

The grouped ASSR amplitudes were found to be significantly different (larger) in the group with poor speech comprehension for the mean ASSR response in the quiet condition (Figure 6B, top panel, *p* = 0.037) and in ipsilateral noise masking for 4 kHz (Figure 6B, lower panel, *p* = 0.045). Also, in the post hoc linear-mixed model analysis after permutation, ASSR was the only electrophysiological measure that significantly explained a considerable amount (7%) of speech-in-noise comprehension (*p* = 0.012); this corresponded to 0.4 dB under the broadband condition. Therefore, under quiet and ipsilateral noise conditions, variations in speech comprehension of OLSA-BB normalized for the PTT showed a significant association between larger ASSR amplitudes and poor speech reception thresholds of OLSA-BB (Figure 6B,C, *p* = 0.0037).

In summary: Poor speech comprehension in quiet and noise, which remained when the OLSA threshold was normalized for the PTT, was associated with slightly increased amplitudes of TENV coding, although over age, ASSR amplitudes decreased.

### 3.7. The Difference between Good and Poor PNOT Is Reflected in Differences in Cochlear Amplifier Efficiency at Stimulus Onset

**Figure 7:**
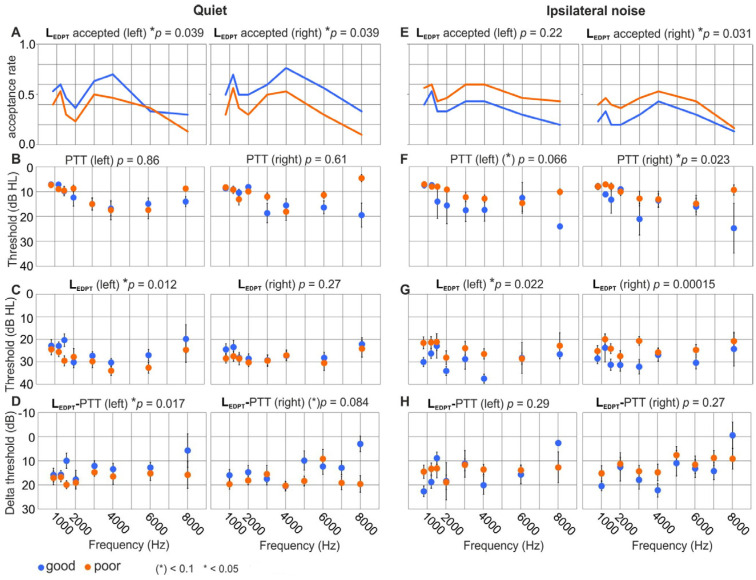
(**A**,**E**) L_EDPT_ acceptance rates, (**B**,**F**) PTT, (**C**,**G**) L_EDPT_, (**D**,**H**) L_EDPT_-to-PTT difference for left and right ears are compared between good (blue) and poor (orange) speech-in-quiet comprehension performers. Participants with good speech-in-quiet performance (blue) showed higher acceptance rates (**A**), equal PTT (**B**), inconclusive L_EDPT_ (**C**), but a consistent 3 dB better threshold for L_EDPT_-to-PTT although on the right ear with only *p* = 0.084 (**D**). Estimated distortion-product thresholds (L_EDPT_) in relation to PTT, when participants are grouped with respect to their speech-in-ipsilateral-noise performance (**E**) L_EDPT_ acceptance rates, (**F**) PTT, (**G**) L_EDPT_, (**H**) L_EDPT_-to-PTT difference for left and right ears are compared between good (blue) and poor (orange) speech-in-noise comprehension performers. Participants with good speech-in-noise performance (blue) show reduced acceptance rates, reduced PTT and L_EDPT_, but no difference for L_EDPT_-to-PTT.

When pDPOAE growth functions were analyzed in participants that were classified by good (Figure 7A–D, blue) or poor (Figure 7A–D, orange) speech-in-quiet recognition, four out of ten factors became significant, and one became a tendency. (i) The percentage of accepted estimates of pDPOAE thresholds (L_EDPT_) was higher for participants with good speech reception in comparison to participants with poor speech reception for both the left (n = 60 ears; *p* = 0.039) and right (n = 60 ears; *p* = 0.039) ear (Figure 7A). The difference in acceptance rate was assessed with a chi-squared test, using the two speech performance groups as the first dimension, and above or below-average L_EDPT_ acceptance rate as a second dimension. (ii) The PTT was not different between groups (Figure 7B). (iii) For the left ear, L_EDPT_ was significantly lower for the good performers (*p* = 0.012) (Figure 7C). (iv) When L_EDPT_ was normalized for the PTT, a significantly lower cochlear threshold persisted in participants with good speech comprehension in comparison to those with poor speech comprehension in the left ear (*p* = 0.017) and remained different with a tendency (*p* = 0.084) for the right ear (Figure 7D). The difference in normalized L_EDPT_–PTT between the groups was 2.8 and 3.1 dB for the right and left ear, respectively (Figure 7D). Even when excluding the results for 8 kHz, higher L_EDPT_–PTT differences were observed for subjects with poor speech performance, showing again significance in the left and a tendency in the right ear. Thus, semi-logarithmic DPOAE I/O functions, a measure that relates to cochlear amplification at near-threshold sound-pressure levels (L_EDPT_), as well as a measure that is influenced by cochlear amplification at stimulus levels up to 55 dB SPL (acceptance rate), represent a stronger cochlear amplifier (lower L_EDPT_ values, higher acceptance rates) if a subject has good speech-in-quiet recognition, or weaker amplifier if a subject has poor speech-in quiet recognition. If one disregards the lack of complete consistency, the conclusion would be that a stronger pre-neural input signal to the IHC is an advantage for speech-in-quiet recognition for subjects with an equal behavioral PTT, and thus could point to a previously unrecognized influence of cochlear amplification in speech reception in quiet.

When pDPOAEs were analyzed in participants that were classified by good (Figure 7E–H, blue) and poor (Figure 7E–H, orange) speech-in-ipsilateral-noise recognition, five out of ten factors became significant, and one a tendency (only four measures for the two ears shown in Figure 7; we omitted the slope of the I/O functions that, with one exception, never became significant). In contrast to quiet conditions, the acceptance rate was significantly higher for poor performers in the right ear (*p* = 0.031) (Figure 7E), and the PTT was lower for poor performers, with a tendency in the left ear (*p* = 0.066), and significant in the right ear (*p* = 0.023; Figure 7F). Moreover, L_EDPT_ was significantly lower for poor performers in the left (*p* = 0.0022) and the right ear (*p* = 0.00015, Figure 7G). In addition, the slope in the right ear was significantly steeper for poor performers (*p* = 0.041, not included in Figure 7). Thus, measures of hearing sensitivity close to the threshold, the PTT, the distortion-product threshold (L_EDPT_), and a measure that is at least influenced by cochlear amplification at levels up to 55 dB SPL (acceptance rate) represent stronger cochlear amplification (lower L_EDPT_ values, lower behavioral thresholds, higher acceptance rates) if a subject has poor speech-in-noise recognition.

This supports the finding that, in contrast to subjects with poor speech comprehension in quiet, those with poor speech comprehension in noise do not have low, but rather higher, cochlear-amplification performance.

Finally, we tested whether there were contributions to the total variance of speech comprehension performance based on each of the three differently filtered versions of OLSA. We found that the DPOAE I/O function acceptance rate (Figure 7A), as well as the difference between L_EDPT_ and PTT (Figure 7D), survived the most restrictive post hoc linear mixed-model analysis after permutation (*p* = 0.001–0.033), explaining 2.0–8.3% of the variance, or 0.8 dB, and 3.2–4.8 dB of the SRT_50_ variation in the broadband and high-pass condition, respectively (Supplementary Figure S1, Supplementary Table S4). Under ipsilateral noise, the acceptance rate of L_EDPT_ measurements (Figure 7A) was significant to explain the variance of SRT_50_ in broadband and high-pass condition and almost significant (*p* = 0.051) in the low-pass condition, accounting for 3.1 to 5.5% of the variance, but only 0.3 to 1.0 dB in SRT_50_. Here, the PTT-corrected L_EDPT_ thresholds explained up to 7% (0.3 dB in SRT_50_) of the variance of the OLSA, but only in the low-pass condition (Supplementary Figure S1, Supplementary Table S3).

In summary: Poor speech comprehension in quiet, independent of PTT and age, is linked to elevated pDPOAE thresholds, putatively reflecting a poorer pre-neural input signal at stimulus onset. Poorer speech comprehension in noise, in contrast, is linked with lower pDPOAE thresholds, thus reflecting rather stronger pre-neural input signals at stimulus onset.

### 3.8. The Difference between Good and Poor PNOTs Is Reflected in Variations in Supra-Threshold Amplitude and Response Latencies of ANFs

**Figure 8:**
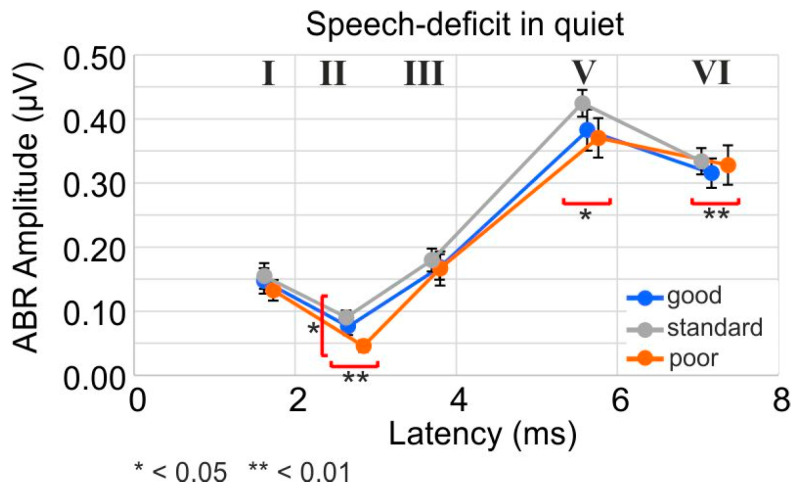
ABR wave amplitude as a function of ABR wave latency in participants matched for PTA thresholds and grouped for good (blue), standard (grey), or poor (orange) speech comprehension in quiet. Significant shifts in latency in poor comprehension, in comparison to the group with good speech comprehension were observed (ABR wave I latency: n = 29, 27, 24, *p* = 0.218242; wave II latency: n = 24, 22, 16, *p* = 0.007707, wave III latency: n = 30, 28, 26, *p* = 0.182784; wave V latency: n = 30, 28, 28, *p* = 0.026617 and wave VI latency: n = 27, 27, 24, *p* = 0.001055).

Lower efficiency of cochlear amplification, resulting in a poorer pre-neural input signal at stimulus onset, as observed here in individuals with poor speech comprehension in quiet, may influence the fast onset peak of the ANF spike rate that contributes to ABR peak amplitude. This reflects the peak spike-rate increase before the ANF firing rate declines to a steady-state value [84]. To measure this, we analyzed the amplitudes of supra-threshold ABR waves for PNOT groups (Figure 8). We observed that the amplitude of wave I differed by 0.0148 +/− 0.0129 µV in participants with poor speech comprehension, in comparison to those with good speech comprehension (Figure 8). This limits the detection threshold for input amplitude differences to 5 dB when assuming the ABR wave I growth with respect to the sound presentation level, as described by [85]. Moreover, we found a significantly smaller ABR wave II amplitude in participants with poor speech comprehension in comparison to those with good speech comprehension (Figure 8, good: 0.0767 µV, n = 24; standard: 0.0905 µV, n = 22; poor: 0.0458 µV, n = 16; *p* = 0.0458), while no difference was observed in the ABR wave III, V, or VI amplitude between participants with good and poor speech comprehension (Figure 8).

Correspondingly, when testing the predictive ability of ABR waves for variance of speech reception thresholds in quiet (Supplementary Figure S1, Table 1), we found that the ABR wave I amplitude was negatively correlated with the PNOT in OLSA-BB and OLSA-LP condition, explaining 2.5 and 2.8% of the remaining variance, respectively. This means that smaller early supra-threshold ABR wave peak amplitudes reflect poor PNOTs (Figure 8, wave I, II). 

In addition, significant latency shifts were observed in participants with poor speech comprehension in comparison to those with good or standard speech comprehension, as shown for wave II (Figure 8, II, good: 2.66 ms, n = 24; standard: 2.63 ms, n = 22; poor: 2.85 ms, n = 16; *p* = 0.00771), wave V (Figure 8, V, good: 5.63 ms, n = 30; standard: 5.56 ms, n = 28; poor: 5.76 ms, n = 28; *p* = 0.027), and wave VI (Figure 8, VI, good: 7.17 ms, n = 27; standard: 7.04 ms, n = 27; poor: 7.37 ms, n = 24; *p* = 0.0011). It is important to note that these latency differences in the good and poor PNOT groups in quiet did not differ by age (Table 1), or by PTA-EHF (Table 1), and thus exist in addition to the observed age-dependent supra-threshold amplitude and latency differences (Figure 3). Delayed ABR latencies of wave I and V survived the most restrictive post hoc linear mixed-model analysis after permutation (*p* = 0.004–0.030), and significantly explained 2.2% and 2.2% of OLSA-BB variance, and 4.6% and 3.2% of OLSA-LP variation in quiet; this, however, only corresponded to 0.8 to 1.3 dB of the SRT_50_ variation. ABR wave VI latency explained 4.2% of the OLSA-HP in quiet (Supplementary Figure S1, Supplementary Table S3).

In summary: Poor speech comprehension in quiet that remained when OLSA thresholds were normalized for PTT was linked to significantly delayed supra-threshold ABR I–VI peak amplitudes, and slightly smaller early ABR wave I–II amplitudes. This indicates that reduced neural response and/or synchronization at stimulus onset contribute to poor speech comprehension in quiet, independent of PTT.

### 3.9. Delta to Poor and Good PNOTs Show Differences in Phoneme Discrimination below and above the PLL

Aiming next to provide insight if poor and good speech comprehension independent of PTT and age may be linked to a difference in TFS or TENV coding, we presented phoneme pairs with formant contrasts below the PLL requiring TFS coding (/o/-/u/ and /du/-/bu/) or above the PLL requiring TENV coding (/i/-/y/ and /di/-/bi/; Table 2).

**Figure 9:**
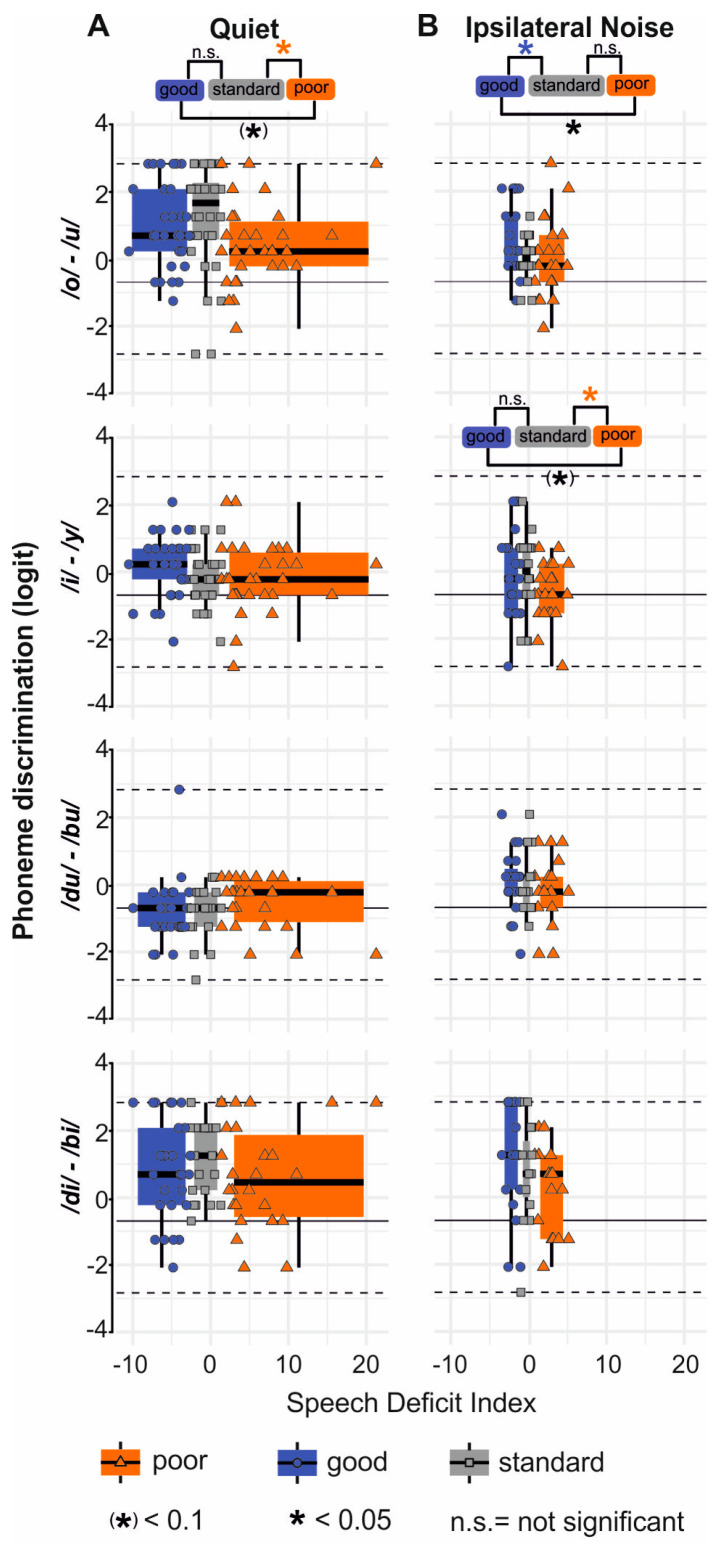
Syllable-discrimination scores in relation to speech comprehension. The scores for four pairs of phonemes (/o/-/u/, /i/-/y/, /du/-/bu/, /di/-/bi/) are segregated for participants with poor (orange), good (blue), and standard (grey) speech comprehension selected by PNOT in quiet (**A**), and ipsilateral noise (**B**). Each plot consists of a boxplot with perceptual performance [% correct] as a function of PNOT (x-axis). Finally, there is a graphical representation of the significance assessed by Mann–Whitney U tests (Supplementary Table S4), significant differences are shown as asterisks with a color code reflecting the three groups.

All phoneme pairs were presented in randomized blocks to the right ear in quiet or ipsilateral noise (Figure 9). For all tested phoneme pairs, two grades of difficulties were chosen, depending on the size of the physical contrast (here, labeled as “difficult” and “easy”). When the discrimination ability in percent was plotted as a function of age for phoneme-pair discrimination in quiet and ipsilateral noise conditions, a weak correlation was found in the quiet condition for /di/-/bi/ and in the ipsilateral noise condition for /du/-/bu/. 

The ability to discriminate between the phoneme pairs in quiet and ipsilateral noise was plotted against the PNOTs obtained from the corresponding groups (Figure 9). Both easy and difficult discrimination conditions were averaged. Supplementary Table S4 provides statistics on behavioral accuracy.

In general, the performance of all participants was better for the discrimination of /di/-/bi/ than of /du/-/bu/. Thus, the phoneme /du/-/bu/ (Figure 9A,B, /du-bu/) showed the smallest variation in behavioral results across the cohort, with performance exceeding the 66th percentile only for a single participant (CS083). This was regardless of age; on average 29.4% to 58.9% of the participants responded below or at the 33rd percentile mark (=chance level), depending on the noise condition. The highest percentage of correct behavioral responses was achieved for discrimination of /o/-/u/ and /di/-/bi/, less for /i/-/y/ (Figure 9A,B).

The most prominent difference between participants with good or poor PNOT was in the differentiation of /o/-/u/, with formant contrasts below the PLL, as shown for quiet (Figure 9A), and ipsilateral noise (Figure 9B). When PNOT categorization in quiet (Figure 9A) was analyzed for phoneme discrimination, it became evident that the /o/-/u/ discrimination performance in participants with poor speech comprehension in quiet was poorer than in the group with standard speech comprehension (Figure 9A). Even under easy conditions, in which the two stimuli had large spectral differences, groups with poor speech comprehension in quiet performed worse in comparison to those with good or standard speech comprehension in quiet (Supplementary Table S4). On the other hand, participants with good speech comprehension (categorized from PNOT) in quiet (Figure 9A, /i/-/y/) were significantly better in their discrimination of /i/-/y/ in comparison to participants with standard speech discrimination ability.

Under ipsilateral noise conditions, participants with good speech comprehension (categorized from PNOTs) were better able to discriminate between /o/-/u/ than participants with standard speech comprehension. However, groups with poor speech comprehension were poorer at discriminating between /i/-/y/ in comparison to those with standard speech comprehension (Figure 9B, /i/-/y/) for both easy and difficult discrimination conditions in ipsilateral noise (Supplementary Table S4).

The discrimination of /du/-/bu/ was not different between participants with good and poor speech comprehension (categorized from PNOTs) in quiet and ipsilateral noise (Figure 9A,B, /du/-bu/), likely because the performance rate among all participants almost never exceeded 60%.

Further, groups with good, standard, and poor speech comprehension (categorized from PNOTs) did not differ in their discrimination ability between /di/-/bi/ under any of the listening conditions (Figure 9A,B, /di/-/bi/), likely because the performance rate among all participants almost always exceeded 90%.

**Figure 10:**
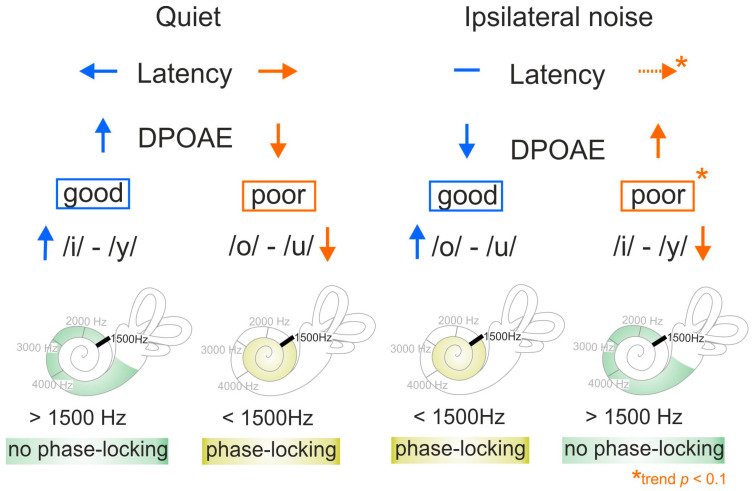
Good and poor speech comprehension in quiet differs from good and poor speech comprehension in ipsilateral noise in the discrimination ability of formant contrasts below PLL (requiring TFS coding), and above PLL (requiring TENV coding). In quiet, poor speech comprehension is associated with poor discrimination below the PLL (e.g., for /o/-/u/), while good speech comprehension is associated with good discrimination above the PLL (e.g., for /i/-/y/). In ipsilateral noise, poor speech comprehension is associated with lower performance for discriminating phoneme pairs with formant contrasts above PLL (/i/-/y/, above 1500 Hz), while good speech comprehension is associated with good discrimination of formants below the PLL (/o/-/u/, below 1500 Hz).

Overall, our findings show that good and poor speech comprehension in quiet differs from good and poor speech comprehension in ipsilateral noise in its discrimination ability of formant contrasts below the PLL (requiring TFS coding), and above the PLL (requiring TENV coding) (Figure 10).

In quiet, poor speech comprehension was associated with poor discrimination of phoneme pairs with formant contrasts below the PLL (/o/-/u/), while good speech comprehension was associated with better discrimination of phoneme pairs with formant contrasts above the PLL (/i/-/y/) (Figure 10, quiet).

In ipsilateral noise, poor speech comprehension was associated with poorer discrimination of phoneme pairs with formant contrasts above the PLL (/i/-/y/), while good speech comprehension was associated with good discrimination of phoneme pairs with formant contrasts below the PLL (/o/-/u/) (Figure 10, ipsilateral noise).

Finally, the differentiation of consonant-based phoneme contrasts that required TFS coding (i.e., /du/-/bu/) was too difficult for both good and poor PNOT groups, while the phoneme contrasts that required TENV coding (i.e., /di/-/bi/) were too easy for both good and poor PNOT groups. Neither of these stimulus pairs therefore resulted in any group differences. Thus, the dynamic range of the phoneme task as implemented here was insufficient for differentiating the influence of PNOTs for /du/-/bu/ and /di/-/bi/ for the different speech-coding mechanisms (TFS vs. TENV).

## 4. Discussion

The present study investigated contributing factors of SRT_50_ for young, middle-aged, and older participants with mostly normal hearing, or mild hearing loss up to 8 kHz. We found that the PTT, although it was the most dominant factor for SRT_50_, only explained approximately half of the variance in quiet and in noise. The variance around this dominant relationship between the PTT and SRT_50_ was then operationally split into three groups of relatively good, standard, and poor speech comprehension, after removing PTT influence by the PNOT method. By analyzing the contributions to the remaining variance (pDPOAEs, ASSRs, supra-threshold ABR wave analysis), a previously undescribed influence of cochlear amplifier efficiency and effectivity and/or synchronicity changes at stimulus onset can be described as contributing to good and poor speech understanding in quiet and noise, regardless of hearing threshold and age. Hair-cell transmission weakness at the beginning of the stimulus should be urgently considered in future for inclusion in clinical diagnostics as a possible cause of speech intelligibility deficits in the young and old.

### 4.1. PTTs and SRT_50_ Show Age-Dependent Differences

In line with previous studies [13,36,86], we observed minor hearing loss in lower frequency ranges (PTA4 and PTA-LF) and prominent hearing loss at HF and EHF (PTA-HF and PTA-EHF) with increasing age (Figure 1). Also, using a differently filtered OLSA spectrum for testing speech comprehension in quiet and ipsilateral noise conditions, in analogy to [40,66] (Figure 2), we here showed speech intelligibility to depend significantly on age under all conditions. Particularly high-pass filtering of the speech material at 1.5 kHz (OLSA-HP) led to the steepest dependence on age (Figure 2). Although frequency spectra beyond 8 kHz have traditionally been noted to exhibit a limited perceptual role in speech sound quality [87,88], evidence is accumulating that high-frequency energy provides at least non-qualitative perceptual information, including cues for speech-source localization and intelligibility (reviewed in [34,36]). From the present study, we cannot exclude the possibility that the considerable loss of PTA-EHF over age (Figure 1) and the strong influence of age on the HP-filtered OLSA spectrum (Figure 2) are related events, and elevated PTA-EHF negatively impacts speech comprehension through lowering the late supra-threshold ABR wave, as discussed below.

### 4.2. Supra-Threshold ABR Wave Decrease with Elevated Age-Dependent PTA-EHF

The peak amplitudes of supra-threshold ABR waves are defined through the precise discharge rate of IHCs onto individual ANFs [89] and the precision with which ANFs fire synchronously at the sound onset [90]. The synchronous firing rate at the onset of the stimulus is a feature that critically depends upon the sensitivity of high-spontaneous firing rate (high-SR) low-threshold ANFs, which define latencies and perception thresholds [91,92,93,94]. In contrast, low-spontaneous firing rate (low-SR) high-threshold ANFs contribute little to the synchronization of ANFs [95]. In the present study, ABR wave I amplitudes were found to be reduced in middle-aged and older subjects (Figure 3A), as also observed in previous work [39,66,96], suggesting that an age-dependent synaptopathy exists in humans. In the present study, the central ABR wave amplitude differed between middle-aged and older individuals, being linked to sustained reduced and delayed ABR wave III–VI in older, but not young or middle-aged individuals (Figure 3A). This lack of compensation in the older group could be associated with a considerable loss of PTA-EHF (Figure 3D), but not PTA-4 or PTA-HF (Figure 3B,C). This relates to a specific effect of EHF on the coding of acoustic signals below 8 kHz, particularly if we take filtering of the in-ear-loudspeakers < 8 kHz used in the present study into account (see Section 2.5). Previous findings analyzing frequency-following responses (FFR) in humans observed a moderate influence of PTA-EHF on lower-frequency changes. Thus, FFR response amplitudes that are the periodic responses to the TFS of frequencies < 1.5 kHz of pure tones, thus below the PLL [97], were negatively influenced by elevated PTA-EHF [79]. While deficits in age-dependent temporal resolution are expected to typically diminish phase-locking at higher stimulation rates [98,99], the present (Figure 2 and Figure 3) and previous [79] findings suggest that in humans, EHF hearing loss may impact synchronous activity at lower frequencies. As low-SR high threshold fibers hardly contribute to the synchronicity of ANFs [95], and OHC dysfunction had no negative effect on temporal coding when phase-locked ANF responses were measured using FFR protocols [79], we may conclude that high-SR low-threshold ANFs driven by low-frequency tones may contribute to the observed differences in central response amplitudes between middle-aged and older individuals (Figure 3A). It remains to be clarified in future studies whether elevated PTA-EHF can influence the transformation of high-SR low-threshold ANFs to a nominal low-SR high-threshold ANF phenotype, as predicted when acoustic overexposure damage of stereocilia contributes to raised thresholds [100]. This aspect should also be taken into account in other studies that describe the influence of PTA-EHF on speech comprehension [101].

### 4.3. Difference between Good and Poor PNOTs Is a Better Indicator of Self-Assessed Hearing Ability than Age

Although SRTs in quiet strongly depend on PTTs, variance analysis in the present study indicates that 38.7% of the variance of OLSA-BB remained unexplained, which corresponds to an SD of 3.7 dB in SRT_50_ (Supplementary Figure S1). SRT_50_ in ipsilateral noise also depends on PTTs, but leaves more of the variance unexplained (Supplementary Figure S1, i.e., 51.4%, corresponding to an SD of 1.0 dB). When speech comprehension differences were normalized for PTT, subjects could be grouped into three categories based on their PNOT (poor, standard, and good). Interestingly, this PNOT categorization revealed a better association between the self-reported evaluations of speech comprehension ability when listening to speech in quiet than when subjects were categorized by age (Figure 5). Although, this correlation was only a statistical tendency, it strengthens previous findings of no correlation between self-reported speech comprehension ability and age [102]. This, moreover, challenges the hypothesis that those factors that influence speech understanding regardless of age and PTT, here identified in the broadest sense as strength or weakness of sound transfer at stimulus onset, may have relevance for the self-assessment of how (well) we hear.

### 4.4. Difference between Good and Poor PNOTs Show Low Dependence on Temporal Coding (ASSR)

As found in previous studies [103,104], the present finding confirmed that the ASSR amplitude declined with age, although only showing a statistical tendency (Figure 6A). Individuals with poor or good speech comprehension independent of PTT appear equally distributed over age (Figure 6A, orange and blue dots). On the other hand, higher ASSR amplitudes were found in poor PNOTs in quiet and ipsilateral noise conditions (Figure 6B) and higher ASSR amplitude in poor PNOTs strongly correlated with OLSA SRT in ipsilateral noise (Figure 6C). Considering an explanation for this surprising finding, we note that the ASSR growth function is known to correlate well with loudness [43,105,106]. This may suggest that our data are driven by a subgroup of poor performers who show extraordinarily high ASSR amplitudes (Figure 6B) and a significant increase in UCL (Supplementary Figure S1), thus exhibiting maladaptive loudness sensation. Here, the underlying mechanism may be linked to a change in the compressive nonlinearity that was suggested to contribute to categorical differences in loudness scaling with steeper growth in loudness for older adults [107], and here possibly to differences in cochlear amplifier efficiency observed in poor PNOTs in ipsilateral noise conditions, as discussed next.

### 4.5. Differences between Good and Poor PNOTs Are Reflected in Variations in Cochlear Amplifier Efficacy at Stimulus Onset

Poor and good speech comprehension, independent of age and PTT, differed between L_EDPT_ and PTT (Figure 7), explaining 2% and 8.3% of the variance in OLSA-BB and OLSA-HP, respectively. A plausible reason for this effect is that L_EDPT_, which is in general closely related to the PTT, is not subject to adaptation of the ANF firing rate [108] or to adaptation caused by the medial olivocochlear reflex, since its time constant [109,110] is well above the DPOAE stimulus pulse widths used here for pDPOAE growth functions. As mentioned before, PTTs as implemented in clinical audiometry are effectively integrated over ~500 ms, reflecting the adapted state of nerve firing [111], while pDPOAEs would rather provide information on the non-adapted, pre-neural input signal to the IHCs [44]. In mammalian IHCs, weakness of nerve adaptation at the onset is associated with vesicle depletion, and is characteristically linked to synaptic fatigue or the desensitization kinetics of postsynaptic receptors [111,112,113,114]. Thus, a larger or smaller L_EDPT_–PTT difference that correlates with better or worse speech-in-quiet comprehension (Figure 7A,D) would reflect stronger or lower firing-rate adaptation, linked to less or more synaptic fatigue at IHCs. These are factors that now need to be considered as a plausible mechanism for differences in the detection of signal-onset features during speech presentation.

In the current data, L_EDPT_–PTT and the acceptance rate of DPOAE I/O functions under ipsilateral noise conditions (Figure 7, Supplementary Table S3) explained 7.0% and 5.5% of the remaining variance of SRT_50_ in the OLSA-LP condition, respectively corresponding to 0.3 dB in SRT_50_ for both measures, and explaining 0.7 and 3.1% in the OLSA-HP condition in quiet, respectively, corresponding to 0.5 and 1.1 dB in SRT_50_. The sign of the variance in L_EDPT_–PTT and the acceptance rate switched in ipsilateral noise in comparison to the quiet condition (Supplementary Figure S1, Supplementary Table S3), indicating that in ipsilateral noise, the larger DPOAE I/O function acceptance rate and larger L_EDPT_–PTT are linked to poorer speech comprehension (Figure 7E–H). This finding might be explained by compression of the cochlear input signal to the neural system. For the speech-in-noise test, temporal information is only used within a narrow dynamic level range. If, then, the DPOAE I/O function acceptance rate is comparatively poorer, it would be at levels above which basilar-membrane compression basically ends, and the growth behavior approaches linear dependency, meaning growth behavior would start at lower levels. This may be, on one side, an advantage, because a larger part of the dynamic level range used in the test would be almost linear, yielding uncompromised modulation contrast of the speech signal, as also previously discussed [29]. On the other side, this phenomenon, known as recruitment [115,116], shapes not only DPOAE I/O functions [117] or loudness scaling [107], thereby providing a rationale for the larger ASSR and UCL responses observed here, but also limits high TENV coding as discussed later.

### 4.6. Differences in Good and Poor PNOTs Are Reflected in Variations in Supra-Threshold Amplitude and Response Latencies of Auditory Nerve Fibers

Strikingly, a functional synaptopathy was evident as an ABR wave I amplitude reduction and an ABR wave II–VI latency shift in participants with poor PNOTs in quiet (Figure 8), which were of similar age to those with good PNOTs in quiet groups (Table 1). This factor explained 2.8%–1.0 dB—of OLSA-LP (ABR wave I) and 4.6%–1.3 dB—of OLSA-LP (ABR wave I latency), when SRT_50_ was corrected for PTT (Supplementary Table S3). Independent of OHC loss, which is expected to define the PTT, cochlear synaptopathy is not expected to explain more than 1 dB of OLSA. Indeed, a model of the effects of synapse loss on basic perceptual tasks, which calculated the effects of more than 50% loss of synapses, came up with a just-noticeable difference of up to 1.4 dB [118,119]. In contrast to previous findings reviewed by [120] that argued against a direct role for cochlear synaptopathy in the coding of moderate-to-high-level speech sounds, or assumptions that speech comprehension deficits in quiet are mainly linked to an increase in hearing thresholds [28], the present findings provide strong evidence that cochlear synaptopathy in humans exists even independently of age and PTT, as shown through reduced early and delayed early and late ABR waves in poor PNOTs (Figure 8). The peak amplitudes of supra-threshold ABR waves are defined through the precise discharge rate of individual auditory fibers [89] and the precision with which auditory fibers fire synchronously at the onset [90]. Synchronous firing at the onset of the stimulus is a feature that is critically dependent on the sensitivity of high-SR low-threshold ANFs, which not only define latencies and perception thresholds [91,92,93,94], but through its specific contribution to the rise in spike rate at the onset of sound stimulation [121,122], also define synchronized ANF responses at stimulus onset [95,123]. Therefore, differences in early ABR wave I/II amplitudes and late ABR wave latencies, as observed between subjects with poor and good PNOTs in quiet (Figure 8), may be best explained by differences in high-SR ANF functions at stimulus onset that influence speech comprehension due to an altered impact on synchronized ANFs at stimulus onset. In conclusion, as discussed for the lower pDPOAE acceptance rate in poor PNOTs, a weaker firing-rate onset peak as a result of pre- or postsynaptic changes linked to synaptic fatigue or a desensitization kinetic at IHCs [111,112,113,114] should be discussed as a novel contributor to speech comprehension in quiet, independent of age and PTT.

### 4.7. Differences in Good and Poor PNOTs Are Reflected in Variations in Phoneme Discrimination below and above the PLL

As a most striking feature of the phoneme discrimination test in PNOT groups, we observed poorer speech coding below the PLL in poor PNOT in quiet. Previous findings suggested a crucial role of high-SR ANFs for a perceptional threshold in the phase-locking range [56,124], which must be seen in connection with poorer speech coding below the PLL (Figure 9A, /o/-/u/, Figure 10, Supplementary Table S4). The poor discrimination of phonemes with formant contrasts above the PLL (/i/-/y/) in subjects with poor vs. standard PNOT (Figure 9) is best explained through the higher acceptance rate and L_EDPT_–PTT difference in this group (Figure 7E,H). This is likely linked to the lower basilar-membrane compression, and subsequent diminished width of the dynamic range of low- and medium-SR fibers, required for TENV coding [6], which provides a disadvantage for the differentiation of formant contrasts above the PLL (Figure 9, Supplementary Table S3, Figure 10).

## 5. Conclusions

In conclusion, apart from the dominating threshold dependence evidenced by PTTs, and typically used in clinical routine, we discovered several effects that affect speech discrimination independently of PTTs, which differ depending on whether the speech signal is close to threshold (speech-in-quiet), or clearly supra-threshold (speech-in-noise). Thus, using diagnostic procedures that enable the detection of changes in auditory processing at the beginning of the stimulus, such as pDPOAEs, ABR peak amplitudes, the phoneme discrimination test, and ASSRs as a metric of temporal coding, we identified elements that contribute to speech comprehension independent of age and PTT. As new factors contributing to speech comprehension in quiet, by comparison between DPOAEs and PTTs, we here identified the state of the cochlear amplifier and high-SR cochlear synaptopathy, influencing synchronized ANF responses at stimulus onset.

In noise, it appears that the recruitment phenomenon can partially counteract the discrimination deficits brought about by hearing loss due to reduced cochlear amplification. Differences in the nerve adaptation rate at stimulus onset in quiet and the recruitment phenomenon in noise must therefore be re-included in the 50% differences in human speech understanding that were previously not explained by hearing thresholds, and that likely contribute to the predicted 80% of auditory information that is transmitted at stimulus onset during speech [58]. These elements should be considered as a new mechanism behind the different coding principles predicted to be dependent on the PLL [40,57]. The findings also emphasize the need for improved routine clinical techniques to diagnose sound processing at stimulus onset.

## Figures and Tables

**Table 1:** Pure-tone threshold-normalized SRT_50_ (PNOT) differentiated for the noise condition and the three speech comprehension groups.

Noise Conditions	PNOT	*n*	Age Mean ± SEM	PTA4 Mean ± SEM	PTA-EHF Mean ± SEM
In quiet	good	30	45.40 ± 2.87	12.33 ± 1.21	36.22 ± 3.24
	standard	29	38.48 ± 3.10	13.41 ± 1.44	28.67 ± 3.73
	poor	30	47.60 ± 3.25	13.40 ± 1.18	38.28 ± 3.73
In ipsilateral noise	good	21	45.24 ± 3.64	14.02 ± 1.49	36.00 ± 4.19
	standard	21	41.05 ± 3.87	13.21 ± 1.53	29.42 ± 4.67
	poor	21	42.10 ± 3.70	12.38 ± 1.78	34.30 ± 4.25

**Table 2:** Dependence of all OLSA SRT_50_ on their corresponding pure-tone averages. OLSA-BB on PTA4 (Figure 4A,D,G).

		R^2^	*p*	*n*
OLSA quiet	BB over PTA4	0.5363	<0.00001	89
	LP over PTA-LF	0.4574	<0.00001	89
	HP over PTA-LF	0.0388	0.064394	89
	HP over PTA-EHF	0.378	<0.00001	89
OLSA ipsilateral noise	BB over PTA4	0.3111	<0.00001	63
	LP over PTA-LF	0.064	0.045525	63
	HP over PTA-HF	0.1826	0.000478	63
	HP over PTA-EHF	0.1748	0.00065	63

## Data Availability

The raw data supporting the conclusions of this article will be made available by the authors on request.

## Supplementary Materials

The following supporting information can be downloaded at https://www.mdpi.com/article/10.3390/jcm13092725/s1, Supplementary Figure S1: Post hoc analysis of OLSA variance based on a linear mixed model; Supplementary Table S1: Proband information; Supplementary Table S2: Frequencies of the first four formants; Supplementary Table S3: Post hoc analysis of OLSA variance based on a linear mixed model; Supplementary Table S4: *p*-values for syllable discrimination scores for different speech performers. Supplementary Material pDPOAE measurements. References [44,67,68,69,70,71,72,125,126,127,128,129] has been cited in the Supplementary Materials file.
