# Supplementary material for: Neural Adaptation at Stimulus Onset and Speed of Neural Processing as Critical Contributors to Speech Comprehension Independent of Hearing Threshold or Age
Source: J Clin Med. 2024 May 6;13(9):2725. doi: 10.3390/jcm13092725 (PMC11084258; doi:10.3390/jcm13092725)
Supplement: Supplementary file 1 [file jcm-13-02725-s001.zip › jcm-2962886-supplementary.pdf]

Supplementary Material

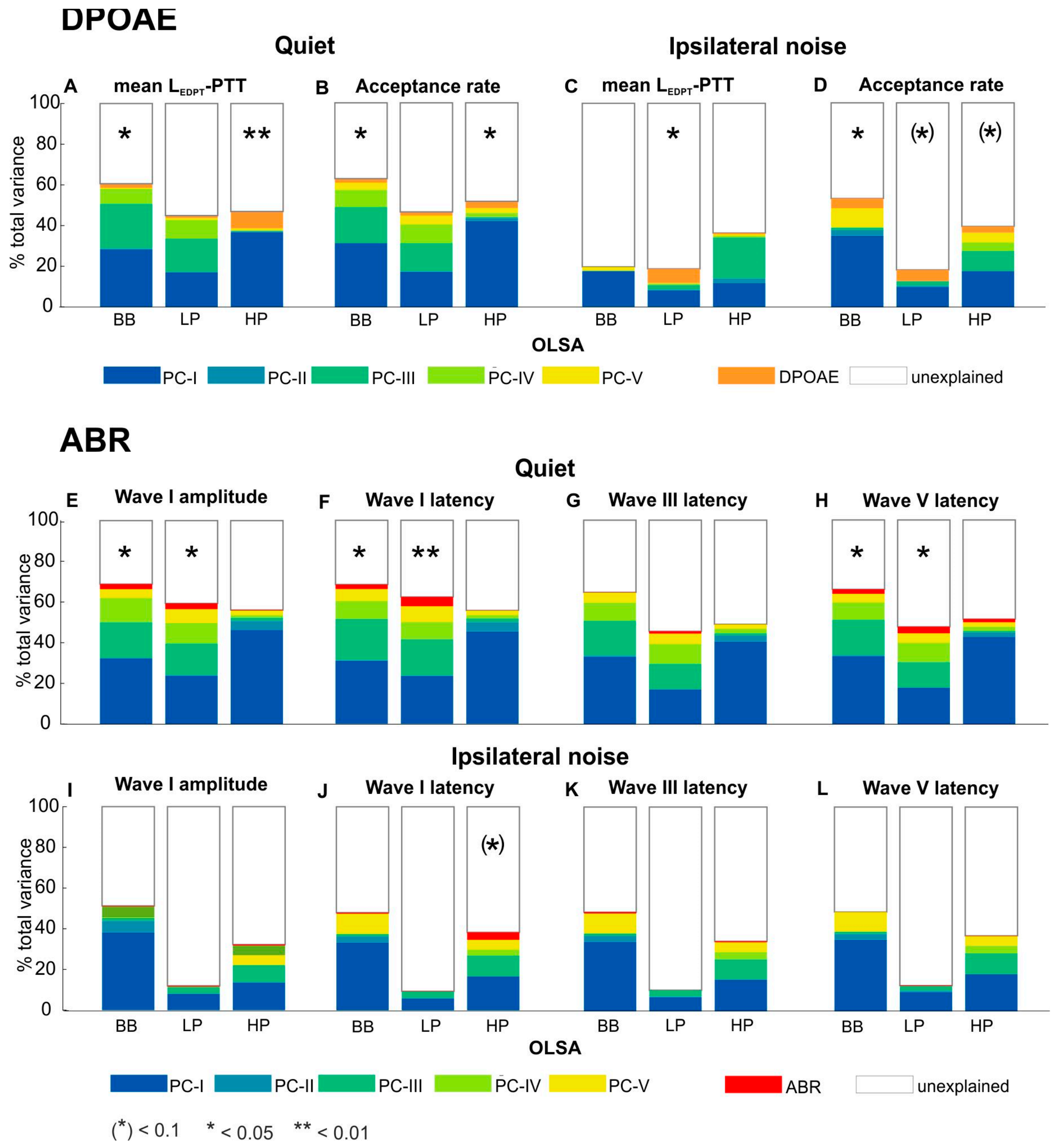

Supplementary Figure S1. Posthoc analysis of OLSA variance based on a linear mixed model **(A-D)** DPOAE: L_{EDPT}-PTT contributing 2% to OLSA-BB and 8.3% to OLSA-HP in quiet **(A)** and 7% (LP) in ipsilateral noise **(C)**. DPOAE acceptance rates **(B, D)** show that the number of valid L_{EDPT} measurements contributing **(B)** 2% OLSA-BB and 3.3% OLSA-HP in quiet and **(D)** 4,8% OLSA-BB in ipsilateral noise. **(E-L)** ABR wave I amplitude **(E, I)** and latencies for wave I **(F, J)**, II **(G, K)** and V **(H, L)** contributing to SRT variance (see Supplementary Table 3). Significance levels based on permutation testing: * p<0.05 and ** p<0.01. Tendencies with p<0.1 are marked with (*).

Supplementary Table S1. Participant information to age, gender (female f, male m), handedness and results from depression evaluation according to Beck Depression Inventory II (BDI-II), Geriatric Depression Scale (GDS) as well as dementia test Mini Mental Status Examination (MMSE) and Tympanometry results.

Proband	Age	Gender	Handedness	BDI*	GDS*	MMSE	Tympanometry
CS001	26	f	right	minimal	minimal	30	Type A
CS002	63	f	right	minimal	minimal	30	Type A
CS003	59	m	right	minimal	minimal	30	Type A
CS004	62	m	right	minimal	minimal	30	Type A
CS005	55	f	right	minimal	minimal	30	Type A
CS006	60	f	right	mild	minimal	30	Type A
CS007	38	f	right	minimal	minimal	30	Type A
CS008	48	f	right	moderate	mild/moderate	30	Type B
CS009	21	f	right	minimal	minimal	30	Type A
CS010	47	f	right	minimal	minimal	30	Type A
CS011	19	f	right	minimal	minimal	30	Type C
CS012	22	m	right	minimal	minimal	29	Type A
CS013	26	f	right	minimal	minimal	30	Type A
CS014	25	m	right	minimal	minimal	30	Type A
CS015	25	m	right	mild	minimal	30	Type A
CS018	24	m	right	minimal	minimal	29	Type A
CS023	69	m	right	moderate	mild/moderate	29	Type A
CS025	64	f	right	minimal	minimal	28	Type A
CS027	60	f	right	minimal	minimal	30	Type A
CS029	64	m	right	minimal	minimal	29	Type A
CS030	23	f	right	minimal	minimal	30	Type A
CS031	53	m	right	minimal	minimal	29	Type A
CS033	26	f	right	minimal	minimal	29	Type A
CS034	24	f	right	minimal	minimal	29	Type A
CS035	45	m	right	minimal	minimal	28	Type A
CS037	45	m	right	minimal	minimal	29	Type A
CS038	57	f	right	minimal	minimal	30	Type A
CS039	18	f	right	minimal	minimal	29	Type A
CS040	57	f	right	minimal	minimal	30	Type A
CS041	58	f	right	minimal	minimal	30	Type A
CS042	54	f	right	mild	minimal	29	Type AS
CS044	62	f	right	minimal	minimal	30	Type AS
CS047	55	m	right	minimal	minimal	29	Type A
CS050	62	f	right	minimal	minimal	29	Type A
CS051	29	m	left	mild	minimal	30	Type AS
CS053	19	m	right	moderate	mild/moderate	30	Type A
CS056	28	f	right	minimal	minimal	30	Type A
CS057	28	f	right	minimal	minimal	29	Type AD
CS058	21	f	right	minimal	minimal	30	Type A
CS059	29	m	left	minimal	minimal	29	Type A
CS060	24	f	right	minimal	minimal	29	Type AD
CS061	58	f	right	minimal	minimal	30	Type A
CS062	52	f	right	minimal	minimal	30	Type A

CS063	28	m	right	minimal	minimal	30	Type A
CS065	23	f	right	minimal	minimal	29	Type A
CS066	70	m	right	minimal	minimal	30	Type AD
CS067	33	m	right	mild	minimal	30	Type AD
CS068	52	f	right	minimal	minimal	30	Type A
CS069	24	f	right	minimal	minimal	30	Type A
CS070	20	f	left	mild	mild/moderate	28	Type A
CS071	60	f	right	minimal	minimal	30	Type A
CS073	43	f	right	minimal	minimal	29	Type A
CS074	21	m	right	moderate	mild/moderate	29	Type A
CS075	54	f	right	minimal	minimal	30	Type A
CS076	30	m	right	minimal	minimal	30	Type A
CS077	52	f	right	minimal	minimal	30	Type A
CS078	35	m	right	minimal	minimal	30	Type A
CS079	34	f	right	minimal	minimal	28	Type A
CS080	35	m	left	minimal	minimal	30	Type A
CS081	54	f	right	minimal	minimal	30	Type A
CS082	48	f	right	minimal	minimal	29	Type AD
CS083	76	m	right	minimal	minimal	29	Type A
CS084	54	f	right	minimal	minimal	30	Type A
CS085	40	f	right	minimal	minimal	28	Type A
CS086	56	f	right	minimal	minimal	30	Type AD
CS087	32	f	right	minimal	minimal	29	Type A
CS088	30	f	right	minimal	minimal	29	Type A
CS089	53	f	right	minimal	minimal	30	Type A
CS090	56	f	right	minimal	minimal	30	Type A
CS091	30	f	right	minimal	minimal	30	Type A
CS093	40	m	right	minimal	minimal	30	Type A
CS095	50	m	right	minimal	minimal	29	Type A
CS096	51	f	right	minimal	minimal	30	Type AS
CS097	40	f	right	minimal	minimal	30	Type A
CS098	54	f	right	minimal	minimal	30	Type A
CS099	27	f	right	minimal	minimal	29	Type A
CS100	25	m	right	minimal	minimal	30	Type A
CS101	46	f	right	minimal	minimal	30	Type A
CS102	21	m	right	mild	minimal	30	Type A
CS103	28	f	right	minimal	minimal	28	Type A
CS104	68	f	right	minimal	minimal	30	Type AS
CS105	65	m	left	minimal	minimal	30	Type A
CS106	23	f	left	minimal	minimal	30	Type A
CS107	64	m	right	minimal	minimal	30	Type B
CS108	70	f	right	minimal	minimal	29	Type A
CS109	70	w	right	minimal	minimal	29	Type A
CS110	72	m	right	minimal	minimal	29	Type AS
CS111	62	m	right	minimal	minimal	30	Type A
CS112	71	f	right	minimal	minimal	29	Type As

Supplementary Table S2. Frequencies of the first four formants (F1 - F4) for each individual vowel sound, measured in Hz.

	/o/	/u/	/i/	/y/
F1	380	200	350	350
F2	750	750	1950	1780
F3	3500	3500	2800	2100
F4	10000	10000	4000	3000

Supplementary Table S3 Posthoc analysis of OLSA variance based on a linear mixed model.

			sign of correlation			Explained total Variance in %			Explain std dB			p value			Total std dB		
		N	bb	lp	hp	bb	lp	hp	bb	lp	hp	bb	lp	hp	bb	lp	hp
LEDPT-PTT	Quiet	81	1	1	1	2.0	1.2	8.3	0.8	0.7	4.8	0.033	0.161	0.001	5.5	6.6	16.6
	Ipsi	56	1	1	1	0.1	7.0	0.7	0.0	0.3	0.5	0.783	0.037	0.519	1.1	1.3	6.0
Number of	Quiet	88	-1	-1	-1	2.0	1.8	3.3	0.8	0.9	3.2	0.037	0.105	0.019	5.9	6.7	17.8
DPOAE	Ipsi	63	1	1	1	4.8	5.5	3.1	0.3	0.3	1.1	0.023	0.051	0.079	1.4	1.3	6.1
ABR amp I	Quiet	76	-1	-1	1	2.5	2.8	0.2	0.9	1.0	0.8	0.022	0.032	0.561	5.4	6.2	16.7
	Ipsi	56	-1	-1	1	0.3	0.3	0.5	0.1	0.1	0.4	0.634	0.671	0.525	1.5	1.3	6.0
ABR lat I	Quiet	80	1	1	-1	2.2	4.6	0.1	0.8	1.3	0.5	0.027	0.004	0.727	5.6	6.3	16.5
	Ipsi	60	-1	1	-1	0.5	0.1	3.7	0.1	0.0	1.2	0.491	0.790	0.063	1.5	1.2	6.0
ABR lat II	Quiet	62	1	1	1	4.0	4.3	2.1	1.0	1.2	2.3	0.018	0.053	0.128	5.2	5.8	16.1
	Ipsi	47	-1	1	-1	0.3	0.2	3.6	0.1	0.1	1.1	0.675	0.745	0.110	1.6	1.2	5.9
ABR lat III	Quiet	84	1	1	-1	0.1	1.1	0.0	0.2	0.7	0.3	0.612	0.211	0.811	5.7	6.7	17.1
	Ipsi	61	1	-1	-1	0.7	0.0	0.5	0.1	0.0	0.4	0.427	0.908	0.487	1.4	1.2	6.0
ABR lat V	Quiet	86	1	1	1	2.2	3.2	1.6	0.8	1.2	2.2	0.024	0.030	0.112	5.6	6.6	17.6
	Ipsi	62	1	-1	-1	0.0	0.3	0.1	0.0	0.1	0.2	0.858	0.667	0.720	1.4	1.3	6.2
ABR lat VI	Quiet	78	1	1	1	1.0	2.0	4.2	0.5	0.9	3.3	0.153	0.105	0.011	5.4	6.7	16.2
	Ipsi	58	-1	-1	-1	0.1	0.4	0.3	0.1	0.1	0.3	0.726	0.613	0.650	1.4	1.3	5.9
ASSR	Quiet	74	1	1	1	0.4	0.1	0.9	0.4	0.2	1.6	0.387	0.749	0.273	5.8	6.8	16.3
	Ipsi	55	1	1	1	7.0	4.3	2.0	0.4	0.3	0.9	0.012	0.109	0.219	1.4	1.3	6.2
ASSR 4k	Quiet	65	1	-1	1	0.0	0.7	0.5	0.1	0.6	1.1	0.875	0.336	0.447	5.9	6.9	15.1
	Ipsi	49	1	1	-1	0.3	0.9	0.1	0.1	0.1	0.2	0.612	0.487	0.805	1.2	1.2	5.9
ASSR 6k	Quiet	68	1	1	1	1.2	0.5	0.2	0.6	0.5	0.7	0.150	0.407	0.632	5.2	6.7	17.7
	Ipsi	50	1	1	1	3.8	0.4	3.3	0.3	0.1	1.1	0.085	0.661	0.147	1.4	1.3	6.3
di bi easy	Quiet	73	-1	-1	1	2.3	1.7	0.2	0.9	0.9	0.8	0.038	0.146	0.603	6.1	7.0	18.2
	Ipsi	51	-1	1	-1	0.1	0.1	0.2	0.0	0.0	0.3	0.761	0.842	0.667	1.4	1.3	6.2
ou difficult	Quiet	88	-1	-1	-1	2.0	0.0	0.2	0.9	0.1	0.7	0.035	0.863	0.603	6.0	6.7	17.8
	Ipsi	62	-1	-1	-1	0.4	1.7	1.6	0.1	0.2	0.8	0.533	0.289	0.216	1.4	1.3	6.2
ou easy	Quiet	88	-1	-1	-1	2.0	0.7	1.2	0.8	0.5	1.9	0.039	0.319	0.169	6.0	6.7	17.8
	Ipsi	62	1	1	-1	0.1	0.0	0.7	0.0	0.0	0.5	0.765	0.899	0.406	1.4	1.3	6.2
iy difficult	Quiet	88	-1	-1	-1	0.6	0.5	0.5	0.5	0.5	1.2	0.256	0.384	0.398	6.0	6.7	17.8
	Ipsi	62	-1	1	-1	4.1	0.1	2.6	0.3	0.0	1.0	0.041	0.748	0.106	1.4	1.3	6.2
iy easy	Quiet	88	1	1	-1	0.0	0.1	0.5	0.1	0.2	1.3	0.892	0.664	0.359	6.0	6.7	17.8
	Ipsi	62	-1	1	-1	1.3	0.3	1.0	0.2	0.1	0.6	0.248	0.680	0.334	1.4	1.3	6.2
UCL 250	Quiet	55	-1	1	1	0.0	0.0	0.1	0.1	0.1	0.6	0.853	0.839	0.729	6.2	7.2	15.9
UCL 250	Ipsi	42	1	1	-1	0.0	10.1	0.7	0.0	0.4	0.5	0.969	0.024	0.497	1.5	1.2	6.2
UCL 500	Quiet	69	-1	1	1	0.0	0.0	1.5	0.1	0.1	2.1	0.863	0.839	0.182	6.2	7.2	17.4

UCL 500	Ipsi	52	1	1	-1	0.3	7.6	0.0	0.1	0.4	0.1	0.619	0.036	0.906	1.5	1.3	6.5
UCL 1000	Quiet	72	-1	-1	1	0.2	0.1	1.9	0.3	0.2	2.4	0.544	0.726	0.117	6.2	7.1	17.8
UCL 1000	Ipsi	53	1	1	-1	0.4	8.6	0.0	0.1	0.4	0.1	0.533	0.022	0.895	1.5	1.3	6.4
UCL2000	Quiet	80	-1	-1	1	0.0	0.1	2.1	0.0	0.2	2.6	0.915	0.776	0.078	6.0	6.8	17.8
UCL2000	Ipsi	56	1	1	-1	0.1	4.4	0.2	0.1	0.3	0.2	0.718	0.099	0.696	1.4	1.3	6.3
UCL4000	Quiet	77	1	1	1	0.1	0.2	1.2	0.2	0.3	1.9	0.692	0.644	0.209	5.9	6.9	17.4
UCL4000	Ipsi	56	1	1	-1	0.3	4.2	0.1	0.1	0.3	0.2	0.572	0.098	0.796	1.4	1.3	6.3
UCL 6000	Quiet	42	1	-1	1	0.1	0.5	1.0	0.2	0.6	1.9	0.782	0.616	0.417	7.1	8.4	19.0
UCL 6000	Ipsi	25	-1	-1	-1	1.2	1.4	0.0	0.2	0.2	0.0	0.455	0.515	0.947	1.4	1.4	5.9

Supplementary Table S4. P-values for syllable discrimination scores for poor, standard, and good speech performers.

listening condition		quiet			ipsilateral noise masking		
group comparison		poor vs. good	poor vs. standard	good vs. standard	poor vs. good	poor vs. standard	good vs. standard
/o/ - /u/	(difficult)	0.059	0.011	0.159	0.032	0.337	0.035
/i/ - /y/	(difficult)	0.496	0.444	0.041	0.081	0.031	0.433
/du/ - /bu/	(difficult)	(0.0314)	(0.0584)	0.334	0.248	0.448	0.233
/di/ - /bi/	(difficult)	0.291	0.102	0.264	0.042	0.187	0.189
/o/ - /u/	(easy)	0.007	0.001	0.264	0.212	0.337	0.125
/i/ - /y/	(easy)	0.014	0.084	0.236	0.129	0.248	0.356
/du/ - /bu/	(easy)	0.076	0.284	0.271	0.179	0.061	0.245
/di/ - /bi/	(easy)	0.264	0.176	0.378	0.284	0.326	0.492

Supplementary material pDPOAE measurements

Short-pulsed distortion-product otoacoustic emissions (pDPOAE) were measured to characterize the pre-neural state of the cochlea. Using a pulsed waveform for the second primary (f_2), along with onset decomposition [1], a technique to capture the short-latency nonlinear-distortion (ND) component of the DPOAE [2], artefactual interference effects with the longer latency component can be safely avoided [3]. Extrapolated DPOAE thresholds based on semi-logarithmically scaled I/O functions have been shown to correlate nearly 1:1 with pure-tone threshold [1,4-6] when stimulus levels of both primaries are chosen according to a so-called scissors paradigm [7]. In contrast, DPOAE amplitudes as a stand-alone measure bear a more complicated, frequency- and level-dependent relationship to behavioral thresholds [1,4-6]. The standard deviation of the residual of the correlation between L_{EDPT} and pure-tone Békésy-tracking audiometry at f_2 has been shown to range between 5 and 8 dB depending on frequency [1]. Most previous studies as e.g [4-7] used several criteria for I/O functions to be accepted, which effectively avoid hard-to-interpret DPOAE I/O functions that lead to large estimation errors at the cost of losing information. Of course, any inter-individual variation in synaptic and neural transmission as well as variation in performing the subjective threshold-detection task contributes to the prediction error. Thus, it is and has been understood that the measured DPOAE threshold is merely a predictor for the state of the cochlear amplifier, or, with other words, a predictor for the pre-neural input signal to the mechano-electrical transduction of the inner hair cells [1]. In the present study pDPOAE input-output (I/O) functions were measured at 8 frequencies (f_2=0.8, 1.2, 1.5, 2, 3, 4, 6, 8 kHz) using an adaptive algorithm comprising at least four pDPOAE values. Estimated distortion-product thresholds (L_{EDPT}) were derived from these ND-DPOAE based on linear regression to the semi-logarithmically scaled ND-DPOAE-I/O functions (p_{DP} [µPa] vs. L_2 [dB SPL], cf. [1,4].

The L_{EDPT} derived from ND-DPOAE I/O functions as well as so-called pDPOAE level maps have been shown to correlate in an almost 1:1 relationship with auditory thresholds with a hitherto exceptionally small standard deviation of the residual of approximately 6 dB [1,8] over a frequency range of at least 1–8 kHz, and to be highly reproducible [9].

pDPOAE were measured unilaterally with ER-10C probes (Etymotic Res., Elk Grove Village, IL). Calibration of the loudspeakers and the microphone included correction for probe-to-tympanic membrane transfer function based on an artificial ear simulator (B&K type 4157, Bruel & Kjær, Nærum, Denmark) and thus are considered to correspond to sound pressure at the tympanic membrane; for details of calibration procedures, see [1]. In order to be accepted, the linear regression to the experimentally obtained I/O function must fulfill several criteria, the most important being related to the quality of the regression procedure, namely regarding the squared correlation coefficient, $r^2_{I/O} \geq 0.8$, and the standard deviation of the L_{EDPT}, $\sigma_{EDPT} \leq 10$ dB (for further details, see [1]. The acceptance rate reported in the Results Section is then the ratio between accepted I/O functions within a group of n_g members across all eight frequencies and both ears, i.e. $n_{acc}/(2*8*n_g)$. Acceptance rate depends on DPOAE amplitudes, background noise, and the linearity of the measured I/O functions, to mention some important contributors. Two sets of interleaved presentations of four two-tone pulse pairs each were averaged over 100 ensembles, where one ensemble consists of four blocks with suitable phase shifts yielding a time-domain DPOAE response with vanishing contribution of the two stimulus tones, when averaged; this method is called primary-tone phase variation technique [10]. The first set comprised presentations with f_2=0.8, 1.5, 3, and 6 kHz in a block of 180 ms length, the second with f_2=1.2, 2, 4, and 8 kHz in a block of 120 ms length. The adaptive procedure started with L_2=45 dB SPL for each f_2-pulse, followed by lower or higher stimulus levels depending on SNR, a minimum L_2 step size of 3 dB, and estimations based on population data of I/O slopes [11]. To be accepted for the subsequent DPOAE threshold estimation, a single ND-DPOAE value requires signal to noise ratios (SNR) ≥ 10 dB. L_1 values are chosen according to a frequency-specific scissor paradigm [1] aimed to equalize both travelling-wave amplitudes of the stimulus tones f_1 and f_2 at the f_2-place in the cochlea (cf. [8]). f_1-pulses were switched on 2.5 ms after corresponding f_2-pulses and presented with widths between 40 to 10 ms adjusted to ensure that intracochlearly the correspondent travelling wave has reached a steady state before the f_2-pulse is switched on. f_2-pulses had half-maximum widths decreasing from 11.9 to 3.0 ms with ramps decreasing from 4.6 to 1.2 ms for frequencies between 0.8 and 8 kHz, designed to roughly allow settling of the ND component of the DPOAE before the second component of the DPOAE, the coherent-reflection component, builds up, so that both components can be visually distinguished in the time domain [12].

Study limits

A limitation to interpreting frequency-specific aspects of DPOAE I/O function acceptance rate in relation to phoneme discrimination is that the speech-intelligibility tests of the BB, LP, and HP conditions were presented at different intensities per frequency band because they were normalized so that the speech stimuli had the same overall perceptual levels. This may be a confounding effect, especially with respect to recruitment.

It is also important to note that the numbers for syllable-discrimination score in Figure 9 and Supplementary Table 4 are not corrected for multiple comparisons, and these effects were likely limited by saturation effects in task performance, resulting in a non-linear dependence, which our linear model of variance analysis may not have been able to capture optimally.

Moreover, limits in exceeding dimensions for statistical analysis allowed the variance analysis for phoneme discrimination only as a linear dependency between good and poor PNOT, but not for the non-linear contribution of phoneme discrimination deficits in participants with good against standard, or poor against standard speech comprehension. Differing from results obtained from non-linear dependence (Figure 9, Supplementary Table 4), the linear dependencies for phoneme contrasts in good and poor PNOT resulted in a significant explanation of variance in quiet for /di/-/bi/ easy by 2.3%, /o/-/u/ difficult 2%, for / o/-/u/ easy 2%, and in ipsilateral noise for /i/-/y/ difficult contributing to 4.1% of variance (Supplementary Table 3).

References Supplementary section

1. Zelle, D.; Lorenz, L.; Thiericke, J.P.; Gummer, A.W.; Dalhoff, E. Input-output functions of the nonlinear-distortion component of distortion-product otoacoustic emissions in normal and hearing-impaired human ears. *J Acoust Soc Am* **2017**, *141*, 3203, doi:10.1121/1.4982923.
2. Shera, C.A.; Guinan, J.J., Jr. Evoked otoacoustic emissions arise by two fundamentally different mechanisms: a taxonomy for mammalian OAEs. *J Acoust.Soc.Am.* **1999**, *105*, 782-798.
3. Zelle, D.; Gummer, A.W.; Dalhoff, E. Extraction of distortion-product otoacoustic emission source components and its relevance for objective audiometry. *Procedia IUTAM* **2016**, doi:10.1121/1.4809772 [doi].
4. Boege, P.; Janssen, T. Pure-tone threshold estimation from extrapolated distortion product otoacoustic emission I/O-functions in normal and cochlear hearing loss ears. *J Acoust Soc Am* **2002**, *111*, 1810-1818.
5. Gorga, M.P.; Neely, S.T.; Dorn, P.A.; Hoover, B.M. Further efforts to predict pure-tone thresholds from distortion product otoacoustic emission input/output functions. *J.Acoust.Soc.Am.* **2003**, *113*, 3275-3284.
6. Johnson, T.A.; Neely, S.T.; Kopun, J.G.; Dierking, D.M.; Tan, H.; Converse, C.; Kennedy, E.; Gorga, M.P. Distortion product otoacoustic emissions: cochlear-source contributions and clinical test performance. *J.Acoust.Soc.Am.* **2007**, *122*, 3539-3553.
7. Kummer, P.; Janssen, T.; Arnold, W. The level and growth behavior of the 2 f1-f2 distortion product otoacoustic emission and its relationship to auditory sensitivity in normal hearing and cochlear hearing loss. *J.Acoust.Soc.Am.* **1998**, *103*, 3431-3444.
8. Zelle, D.; Bader, K.; Dierkes, L.; Gummer, A.W.; Dalhoff, E. Derivation of input-output functions from distortion-product otoacoustic emission level maps. *The Journal of the Acoustical Society of America* **2020**, *147*, 3169-3187, doi:10.1121/10.0001142.
9. Bader, K.; Dierkes, L.; Braun, L.H.; Gummer, A.W.; Dalhoff, E.; Zelle, D. Test-retest reliability of distortion-product thresholds compared to behavioral auditory thresholds. *Hear Res* **2021**, *406*, 108232, doi:10.1016/j.heares.2021.108232.
10. Whitehead, M.L.; Stagner, B.B.; Martin, G.K.; Lonsbury-Martin, B.L. Visualization of the onset of distortion-product otoacoustic emissions, and measurement of their latency. *J Acoust Soc Am* **1996**, *100*, 1663-1679, doi:10.1121/1.416065.
11. Krokenberger, M. Adaptive DPOAE-Wachstumsfunktionen zur objektiven Hörschwellenschätzung bei normalhörenden und hörgeschädigten Ohren. Universität Tübingen, Tübingen, 2019.
12. Zelle, D.; Gummer, A.W.; Dalhoff, E. Extraction of otoacoustic distortion product sources using pulse basis functions. *J Acoust Soc Am* **2013**, *134*, EL64-69, doi:10.1121/1.4809772.
